# Add-on therapy options in asthma not adequately controlled by inhaled corticosteroids: a comprehensive review

**DOI:** 10.1186/1465-9921-5-17

**Published:** 2004-10-27

**Authors:** Hannu Kankaanranta, Aarne Lahdensuo, Eeva Moilanen, Peter J Barnes

**Affiliations:** 1 The Immunopharmacological Research Group, Medical School, University of Tampere, Tampere, Finland; 2 Department of Pulmonary Diseases, Tampere University Hospital, Tampere, Finland; 3 Department of Clinical Chemistry, Tampere University Hospital, Tampere, Finland; 4 Department of Thoracic Medicine, National Heart and Lung Institute, Imperial College, London, UK

**Keywords:** Asthma, inhaled corticosteroids, long-acting β_2_-agonists, theophylline, leukotriene antagonists

## Abstract

Many patients with persistent asthma can be controlled with inhaled corticosteroids (ICS). However, a considerable proportion of patients remain symptomatic, despite the use of ICS. We present systematically evidence that supports the different treatment options. A literature search was made of Medline/PubMed to identify randomised and blinded trials. To demonstrate the benefit that can be obtained by increasing the dose of ICS, dose-response studies with at least three different ICS doses were identified. To demonstrate whether more benefit can be obtained by adding long-acting β_2_-agonist (LABA), leukotriene antagonist (LTRA) or theophylline than by increasing the dose of ICS, studies comparing these options were identified. Thirdly, studies comparing the different "add-on" options were identified. The addition of a LABA is more effective than increasing the dose of ICS in improving asthma control. By increasing the dose of ICS, clinical improvement is likely to be of small magnitude. Addition of a LTRA or theophylline to the treatment regimen appears to be equivalent to doubling the dose of ICS. Addition of a LABA seems to be superior to an LTRA in improving lung function. However, addition of LABA and LTRA may be equal with respect to asthma exacerbations. However, more and longer studies are needed to better clarify the role of LTRAs and theophylline as add-on therapies.

## Introduction

Inhaled corticosteroids (ICS) are the mainstay of current asthma management and should be used in all patients with persistent asthma. Many patients with persistent asthma can be controlled with regular ICS. However, a considerable proportion of patients treated with ICS remain symptomatic, despite the use of low to moderate doses (doses defined according to the ATS classification for adults [1,2]: beclomethasone dipropionate (BDP) 200 – 1000 μg/d, budesonide 200 – 800 μg/d or fluticasone propionate (FP) 100 – 500 μg/d) of ICS. Based on the differences in potency and pharmacokinetics the doses could also be defined differently [3,4]. Recent treatment guidelines [1,2,5,6] classify these patients as having moderate to severe persistent asthma (steps 3 and 4). According to the recent guideline [2] the typical clinical features of step 3 asthma include symptoms daily, nocturnal symptoms at least once a week, exacerbations that may affect activity or sleep, forced expiratory volume in one second (FEV_1_) 60 – 80% of predicted or peak expiratory flow (PEF) between 60 and 80% of the personal best reading. Daily rescue therapy is usually needed. Typical findings include low values of PEF or FEV_1_, a marked variation in daily PEF recordings and/or a significant response to bronchodilators. Thus, asthma is not adequately controlled, and the treatment needs to be optimized.

According to current guidelines the therapeutic options in the treatment of asthma not adequately controlled by low to moderate doses of ICS are as follows: 1. Increase in the dose of the ICS, 2. Addition of long-acting β_2_-agonist (LABA; formoterol or salmeterol), 3. Addition of a leukotriene receptor antagonist (LTRA; montelukast, pranlukast or zafirlukast) and 4. Addition of theophylline. Currently, the National Heart, Lung and Blood Institute guideline [2] recommends addition of LABA as the first choice and gives the other choices as secondary options, but leave the clinician alone to make the decision without offering comprehensive data to support the different options. Recently, this "step-3" dilemma on the different treatment options has gained attention [7,8]. Several of these options have been separately assessed in several reviews, systematic reviews and metaanalyses [7,9-16]. However, no comprehensive reviews exist on the subject. The aim of our article is to review the evidence that supports the increase in the dose of ICS and use of the different "add-on" options. Firstly to demonstrate the benefit that can be obtained by increasing the dose of ICS, dose-response studies with at least three different ICS doses were identified. Secondly, to demonstrate whether more benefit can be obtained by adding LABA, LTRA or theophylline to the treatment than by increasing the dose of ICS, we aimed to identify studies where the addition of a LABA, LTRA or theophylline to the treatment regimen was compared with the addition of a corresponding plabeco to an increased dose (usually doubled dose) of ICS. Thirdly, we aimed to identify studies comparing the different "add-on" options. In this review, we hope to help the clinician facing the "step-3 dilemma" by presenting in a systematic way the evidence obtained from randomised clinical trials that supports the use of these different treatment options.

## Methods

The paper reviews studies where participants were adults or adolescents (≥12 years) with clinical evidence of asthma not adequately controlled with ICS. The general inclusion criteria in this review were: randomized, blinded and controlled trials with either parallel group or cross-over design published as a full-length paper. Steroid-tapering studies were not included as they are difficult to interpret. Studies published in abstract form only were not included. Similarly, studies lasting less than 4 weeks, containing less than 10 patients per group or studies containing a significant proportion (>10%) of patients using systemic steroids were excluded. Similarly "add-on" studies where a significant proportion (>10%) of patients were not using inhaled steroids were excluded.

We made a search of Medline from January 1 1966 to October 2001. All searches were limited to studies published in the English language. To identify the latest studies published, another search was made by using the drug names (budesonide, beclomethasone, fluticasone, flunisolide, mometasone, triamcinolone, formoterol, salmeterol, montelukast, pranlukast, zafirlukast, theophylline) from Medline on October 2003. The searches were manually (HK) evaluated to identify studies fulfilling the inclusion criteria and full papers were retrieved. In the case of uncertainty based on the abstract full papers were retrieved. All studies fulfilling the inclusion criteria for the ICS dose-response studies or "add-on" studies (see below) were scored for quality using the method described by Jadad et al. [17]. Furthermore, relevant systematic reviews were identified from the Cochrane Library (Issue 2, 2003). In addition, some *in vitro *results or results from open, non-randomized or uncontrolled trials or meta-analysis of particular relevance to the present topic may be cited.

### Inclusion criteria for dose-response studies with ICS

To find the dose-response studies with ICS the term "anti-inflammatory agents, steroidal" was combined with the term: "dose-response relationship, drug" (MeSH), which combination produced 249 papers. To demonstrate the dose-response effect of ICS only controlled studies with at least three different ICS doses and a parallel-group design were included. Studies using consecutive doses of steroids were not included because it makes it impossible to differentiate the dose-response relation from the time course relation of efficacy.

### Inclusion criteria for "add-on" studies with long-acting β_2_-agonists, leukotriene antagonists and theophylline

When the basic search done with the term "anti-inflammatory agents, steroidal" was combined with another made with terms: "salmeterol OR formoterol" it produced 97 papers, when combined with a search made with a term "leukotriene antagonists" (MeSH), it produced 26 papers and when combined with a search with a term "theophylline" (MeSH) it produced 342 papers. Only studies where the addition of LABA, LTRA or theophylline to the treatment with inhaled steroid was compared with the addition of a corresponding placebo to an increased dose (usually double-dose) of inhaled steroid were included. In addition, studies comparing the different "add-on" options were identified.

## Increasing the dose of inhaled corticosteroid

### On the design of dose-response studies with ICS

We identified 14 studies [18-31] assessing the dose-response relationship of ICS in the treatment of chronic asthma. All included studies were of fair to excellent quality (Jadad score 3–5). The main characteristics of these studies are presented in **Table 1 **(see Additional file 1). The inclusion criteria in most of these studies were moderate to severe chronic asthma but previous use of small to moderate doses of ICS was not required in all studies. The studies included patients with a relatively wide range of FEV_1 _% predicted and based on that these patients belong to steps 2–4 according to the recent guideline [2]. In all except three studies a ≥12% reversibility in FEV_1 _or PEF in response to a bronchodilator was required. There was 1 study that assessed the dose-response of budesonide, 7 of FP, 1 of BDP, 3 of mometasone furoate, and 2 of triamcinolone acetonide. The studies utilized two main approaches to identify a dose-response relationship. Some studies considered dose-response relationship to be present if the results obtained with the lowest and highest dose of ICS were significantly different, whereas in others the presence or absence of dose-response relationship was characterized with more advanced statistical analysis (e.g. analysis for linear trend or Jonckheere's nonparametric trend test). In this review, both ways of analysis are accepted as evidence for the presence of dose-response. In the following discussion the important difference between the formal dose-response studies presented in this review and the results reported in some meta-analysis is that the data of the meta-analyses may result from studies assessing one or more doses of ICS and comparing their effects with placebo or baseline. Thus, the data derived from some the published meta-analyses [9,11,14,32], although showing a dose-response effect, is obtained by combining different doses from several studies, and is not resulting from a strict dose-response relationship study. In addition, the data obtained using meta-analysis may be derived only from one or two studies.

### Overview on lung function and symptoms in the 14 included studies

Studies with ICS show a statistically significant dose-response effect for morning PEF and FEV_1 _in the treatment of chronic asthma in 9 (69%) and 5 (31%) studies of the 14 studies included, respectively (**Table 2a, **see Additional file 1). However, statistical analysis of dose-dependency fails to show any significant dose-related effect for FVC in 5 (71%) studies of 7 where it was analysed. Similarly, no statistical dose-dependency was found for evening PEF in 6 (50%) studies out of 12 where it was analysed (**Table 2a, **see Additional file 1). The total or daytime symptom scores show a statistically significant dose-response effect in 5 (38%) out of 13 studies, whereas nighttime symptom score showed a dose-dependency in only three (25%) studies out of 13 where it was analysed. A dose-response for the rescue β_2_-agonist use was found in 4 (33%) out of 12 studies where it was analyzed (**Table 2b, **see Additional file 1). The difference between the highest and the lowest dose of ICS was most often statistically significant for morning PEF (7/12 studies; 58%) and to a lesser extent for evening PEF (3/10 studies; 30%), FEV_1 _and total or daytime symptom scores (both 2/12 studies; 16.7%), night-time symptom score and rescue β_2_-agonist use (both 1/11 studies; 9%) and FVC (0/6 studies; 0%). Similarly, the difference between the two consecutive doses of ICS was very seldom statistically significant (**Table 2ab, **see Additional file 1). Thus, taken together, the results suggest that morning and evening PEF and FEV_1 _are more sensitive to show a statistically significant dose-response effect for ICS, whereas symptom scores and rescue β_2_-agonist use are in general less sensitive to the increase in steroid dose. However, this conclusion may also be influenced by the duration of treatment. Inclusion of relatively short studies in this review, may either under- or over-estimate the dose-response differences depending on the outcome measure being used.

#### Beclomethasone dipropionate – studies included in this systematic review

The dose-response relationship of the effects of BDP (100 – 800 μg/d in two different formulations) was evaluated in asthmatic subjects who had deterioration in asthma control after discontinuation of ICS [18]. There was a statistically dose-dependent effect on morning PEF, FEV_1_, FVC, days free from wheeze or chest tightness and β_2_-agonist use, but not on evening PEF or nights free from asthma related sleep disturbance (**Table 2ab, **see Additional file 1). The dose-response effects detected in this study may reflect the fact that the patient population was carefully identified to show a well-defined responsiveness to ICS. Thereafter ICS were withdrawn to induce a clinically meaningful deterioration of asthma control. Thus, the design may not directly reflect clinical practice, where a patient is symptomatic, despite the use of low to moderate doses of ICS.

#### Beclomethasone dipropionate – other literature

A recent meta-analysis [10] analysed the dose-response effect of BDP in the treatment of chronic asthma. Eleven studies with variable methodological quality involved 1614 subjects were included in the analysis. Most of the endpoints were based on only 1–2 studies. In asthmatic patients not treated with oral steroids a small advantage of BDP 800 μg/d over 400 μg/d was apparent for improvement in FEV_1 _and morning PEF and reduction in night-time symptom score compared to baseline. Studies that assessed BDP 1000 v 500 μg/d and BDP 1600 v 400 μg/d demonstrated a significant advantage of the higher dose compared to the lower dose for percentage improvement in airway responsiveness to histamine and FEV_1 _compared to baseline. No differences between higher and lower daily doses of BDP were apparent for daytime symptoms, withdrawals due to asthma exacerbations or oropharyngeal side effects.

#### Budesonide – studies included in this systematic review

A 6 weeks dose-response study in Japanese asthmatics previously not on ICS showed that increasing the dose of budesonide (200–800 μg/d) [19] results in a dose-related improvement in morning and evening PEF and daytime and nighttime symptom scores, but not for FEV_1_. In this study, there was no statistically significant difference between the doubling doses of budesonide (**Table 2ab, **see Additional file 1). Instead, even the lowest dose of budesonide (200 μg/d) was superior to placebo in the case of morning and evening PEF and daytime and night-time symptom scores, but not for FEV_1_.

#### Budesonide – other literature

**Figure 1:**
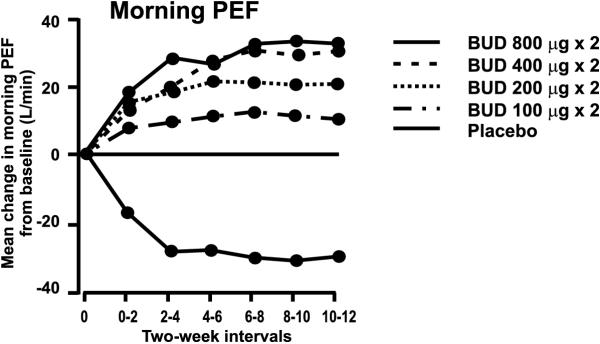
Mean change from baseline in morning peak expiratory flow (PEF) in patients treated with placebo or various doses of budesonide. A significant dose-response effect is seen. However, it should be noted that the difference between placebo and low-dose budesonide is greater than the difference between low-dose budesonide and high-dose budesonide and that there is no statistically significant difference between the various doses of budesonide. Reproduced from reference 33 with permission.

In a randomised, double-blind, placebo-controlled study of parallel-group design lasting 12 weeks four different doses of budesonide (200, 400, 800 and 1600 μg/d were compared in patients suffering from moderate to severe asthma. This study was not included in the systematic analysis due to a high proportion of patients on oral glucocorticoids (15.6%). Increasing the dose of budesonide [33] results in a dose-related improvement in morning PEF and FEV_1_, but not in evening PEF, FVC, symptom scores or rescue β_2_-agonist use. Instead, even the lowest dose of budesonide (200 μg/d) was superior to placebo for all parameters studied. The improvement induced by these low doses is much greater than the difference between the lowest and highest doses of budesonide studied, despite the 8-fold difference in the dose (Figure 1) [33]. There was a statistically significant difference only between the lowest (200 μg/d) and the highest (1600 μg/d) doses of budesonide when morning PEF or FEV_1 _were analysed. Instead, the lowest (200 μg/d) or the highest dose (1600 μg/d) did not differ from the two medium doses (400–800 μg/d). When evening PEF, FVC, daytime or nighttime asthma symptom scores or the use of rescue medication were analysed, there was no significant differences between any of the studied budesonide doses [33].

The dose-relationship of budesonide in the treatment of chronic asthma is a subject of a recent Cochrane review [12]. In this meta-analysis including both children and adults (n = 3907) in non-oral steroid-treated mild to moderately severe asthmatics no clinically worthwhile differences in FEV_1_, morning PEF, symptom scores or rescue β_2_-agonist use were apparent across a dose range of 200–1600 μg/d. However, in moderate to severe asthma there was a significant reduction in the likelihood of trial withdrawal due to asthma exacerbation with budesonide 800 μg/d compared with budesonide 200 μg/d. The reviewers also conclude that budesonide exhibits a significant improvements favouring high dose (1600 μg/d) over low dose (200 μg/d) for improvement in FEV_1 _in severe asthma [12]. Another recent meta-analysis combining 3 placebo-controlled studies with at least two different budesonide doses demonstrated a statistically significant dose-response for morning PEF and FEV_1 _but not for evening PEF [14].

#### Fluticasone propionate – studies included in this systematic review

The dose-dependency of FP has been studied in seven studies in patients with mild to moderate asthma. In two of the studies, patients were previously not on ICS (**Table 1, **see Additional file 1). The difference between the highest and lowest dose was 4- to 20-fold. In all studies almost all parameters improved significantly better with all doses of FP as compared with placebo. Only three studies [20,21,26] show a dose-response effect on morning PEF, only two studies [20,26] show a dose-response relationship for evening PEF and rescue medication use and only one study [20] shows a dose-response relationship for FEV_1_, FVC and daytime symptom score (**Table 2ab, **see Additional file 1). When different doses of FP (50–200–1000 μg/d) were studied in a randomized, double-blind dose-response setting, there was no difference in FEV_1_, FVC, evening PEF, symptom scores, use of rescue medication or the number of night awakenings between the lowest and highest FP dose, despite a 20-fold difference in the dose [21]. Only for morning PEF was the high (1000 μg/d) dose of FP better than the two lower doses, whereas even the lowest dose of FP (50 μg/d) was significantly better than placebo in improving all these parameters.

In a dose-response study [20] with patients with symptomatic chronic asthma (n = 672) patients were randomized to four different doses of FP (100, 200, 400, 800 μg/d). FP improved lung function and symptoms in a dose-related manner. The linear trend for doubling the dose of FP was calculated to be as follows: morning PEF increased 4.3 L/min (95% CI 1.8–6.8) and FEV_1 _increased 0.03 L (95% CI 0–0.05 in two weeks). How does this translate into clinical practice? When assessing a response to a bronchodilator or when assessing a response to inhaled or oral steroid an improvement of 10–20% above the previous values is often considered significant. Thus, in the above study, this would mean >36 L/min increase in morning PEF values. Recently, the average minimal patient perceivable improvements have been estimated as 18.8 L/min for PEF and 0.23 L for FEV_1 _[34]. Based on that the increase in lung function obtained by doubling the dose of fluticasone in the above study seems to be only of very limited clinical benefit.

#### Fluticasone propionate – other literature

In a recent meta-analysis [9] the dose-response relation of inhaled FP in adolescents or adults with asthma in eight studies [n = 2324] employing 2–3 different doses of inhaled FP were analysed. The dose-response curve for the raw data began to reach a plateau at around 100–200 μg/d and peaked by 500 μg/d. A negative exponential model for the data indicated that 80% of the benefit at 1000 μg/d was achieved at doses of 70–170 μg/d and 90% by 100–250 μg/d. A quadratic meta-regression showed that the maximum achievable efficacy was obtained by doses of around 500 μg/d. Another recent meta-analysis [11] of 28 studies with 5788 patients (children and adults) with chronic asthma evaluated the dose-response effect of FP, compared to placebo. Evidence for a dose-response effect was apparent for likelihood of trial withdrawal due to lack of efficacy, change in FEV_1_, morning PEF, evening PEF, nighttime awakening score and physician-rated efficacy. It is important to appreciate that this was only evident when improvements over placebo were compared for the highest dose of FP (1000 μg/d) and lowest dose of FP (100 μg/d). There were no significant differences when any other doses were compared (e.g. FP 200 v 100 μg/d, FP 500 v 200 μg/d, FP 1000 v 500 μg/d). Sixty percent (0.31 L; 95% CI 0.27–0.36 L) of the effect on FEV_1 _with FP 1000 μg/d (0.53 L; 95% CI 0.43–0.63 L) was achieved with tenth of the dose. No dose-response effect was apparent for change in symptom score or for rescue β_2_-agonist use [11]. Another recent meta-analysis from the same authors [32] found a statistically significant advantage of FP 200 μg/d over 100 μg/d for morning PEF (6 L/min; 95% CI 1–10 L/min), evening PEF (6 L/min, 95% CI 2–11 L/min) and night-time awakening score (0.17, 95% CI 0.04 – 0.30), but not for FEV_1_, daily symptom score, night-time awakenings and daily use of rescue β_2_-agonist use. No significant advantage was obtained with the use of FP at doses of 400–500 μg/d over 200 μg/d for morning or evening PEF, FEV_1_, daily symptom score or rescue β_2_-agonist use. Patients treated with higher dose (800 – 1000 μg/d) of FP achieved significantly greater improvements in morning PEF (22 L/min, 95% CI 15–29 L/min) and evening PEF (13 L/min, 95% CI 6–19 L/min) compared to the lower dose (50–100 μg/d). Another recent meta-analysis [14] including eight trials with at least 2 different doses of FP demonstrated a statistically significant dose-response in morning PEF, evening PEF and asthma symptom score but not in FEV_1 _or β_2_-agonist use.

#### Mometasone furoate and triamcinolone acetonide – studies included in this systematic review

Mometasone furoate is a corticosteroid closely related to FP and is being investigated in a dry powder inhalation formulation for the treatment of asthma [35]. Studies with mometasone furoate [27-29] show a dose-related efficacy in the treatment of mild to moderate asthma when morning PEF is analysed (**Table 2a, **see Additional file 1). Interestingly, even doubling doses of mometasone furoate produced statistically significant improvements in morning and evening PEF (**Table 2a, **see Additional file 1) [27-29]. Occasionally, a statistically significant dose-dependency or difference between the highest and lowest dose was found for evening PEF, FEV_1 _or daytime or total symptom score. In contrast, no significant dose-dependency was found for FVC, nighttime symptom score or rescue β_2_-agonist use (**Table 2ab, **see Additional file 1).

Linear trend analyses showed a dose-response for triamcinolone acetonide (TAA) in the treatment of moderate to severe asthma across the dose-range of 150 to 600 μg/d or 200 to 1600 μg/d for most variables in the two studies included in this review (**Table 2ab, **see Additional file 1) [30,31]. Occasionally, a statistically significant difference was reported even between two consecutive doses of TAA. As compared with placebo, therapeutic activity was generally evident at doses of 150–200 μg daily for all variables with significant clinical efficacy demonstrated for all doses.

#### Mometasone furoate and triamcinolone acetonide – other literature

A four-week randomised, double-blind, double-dummy and parallel group study [36] comparing the efficacy and safety of mometasone furoate administered by metered dose inhaler (112, 400 and 1000 μg/d) with BDP (336 μg/d) and placebo recruited adult patients with moderate asthma (n = 395). The patients were required to have a stable ICS dose, FEV_1 _or 50–90% and a bronchodilator response of ≥15% in absolute FEV_1 _at baseline. This study reported significantly better improvement in FEV_1_, FVC and morning PEF with doses of 400 and 1000 μg/d than with 112 μg/d. Also, physician's evaluation of asthma symptoms, but not salbutamol use was significantly better with dose 1000 μg/d than with 112 μg/d. This study, although fulfilling the criteria for dose-response study as defined in materials and methods, was excluded from the systematic evaluation, as the published statistical analysis did not include any formal dose-response analysis, and the reported difference between different mometasone doses always required a statistically significant difference to the active comparator BDP.

In contrast to the results presented in this review (**Table 2ab, **see Additional file 1), a meta-analysis [14] including 2 studies with mometasone furoate (200 μg/d versus 400 μg/d) failed to show any significant dose-response in FEV_1_. In the meta-analysis, there was not enough data to analyse other parameters than FEV_1_. The 3 studies [27-29] included in this review were not included in the meta-analysis [14]. The data suggests that 200 μg/d of mometasone furoate may be a relatively small dose. As both the inhaler device and mometasone have not been available for the treatment of asthma, it is difficult to define their exact position in the treatment of asthma, although there are data to suggest that a total daily dose of 400 μg of mometasone furoate administered with dry powder inhaler may be equal to total daily dose of 500 μg of FP via a Diskhaler or a daily dose of 800 μg budesonide via a Turbuhaler [28,29].

A placebo-controlled, double-blind parallel-group study assessed the effects of three different doses of TAA (450, 900 and 1800 μg/d for 12 weeks; delivered using a non-chlorofluorocarbon propellant) in patients with chronic symptomatic asthma and using ICS [37]. The data for all variables (FEV_1_, FEF_25–75_, morning and evening PEF, symptom scores and rescue salbutamol use) shows that even the lowest dose significantly differs from placebo, and there appears to be no clear dose-response. However, no formal statistical analysis was reported for the presence of a dose-response and thus this study is not included in Tables 1–2. A recent meta-analysis [14] including 3 studies with TAA, demonstrated a statistically significant dose-response in morning PEF, evening PEF and asthma symptom score, but not in FEV_1_.

### Conclusions on the effects of ICS on lung function and asthma symptoms

**Figure 2:**
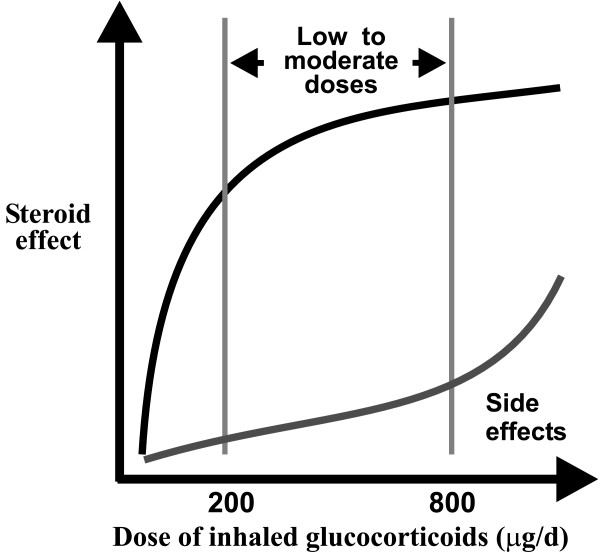
The dose-response curve of inhaled glucocorticoids.

Taken together these results indicate that the change in the ICS dose from low dose to moderate dose is at the flat part of the ICS dose-response curve for most lung function and symptom parameters studied (Figure 2). Furthermore, it appears that the low and moderate doses of currently used ICS are in the flat part of the steroid dose-response curve. Thus, it is predicted that doubling the dose of ICS is not sufficient to significantly improve lung function or reduce symptoms. Rather, the data suggest that the increase in the dose of ICS should be at least 4-fold to produce a clinically significant improvement in variables such as symptoms, use of rescue β_2_-agonists, PEF or lung function. However, the steepness of the dose-response curve for different outcomes may vary. For example, an open dose-response evaluation of different sequential doses of budesonide in patients with mild-to-moderate asthma (38) shows that the dose-response curves for FEV_1_/PEF and FEF_25–75 _are not identical. Similarly, the dose-response curves of budesonide on adenosine monophosphate (AMP) and methacholine bronchial challenges were significantly different [38]. It should also be noted that patients often receive higher doses of ICS in their daily routine treatment than required [3].

The studies discussed above present mean data for groups of patients, but do not address the issue of differences in responsiveness to the anti-inflammatory effects of corticosteroids between individual patients. It may be possible that increasing the dose of ICS may be beneficial for some patients.

### Is there a dose-response in the anti-inflammatory effects of ICS?

#### Studies included in this systematic review

We were not able to identify any studies that would have studied the dose-dependency of the anti-inflammatory effects of ICS in asthma and would have satisfied the inclusion criteria for the present review.

#### Other literature

In a study [39] with patients with chronic asthma (n = 66) treated with moderate doses of ICS the dose-dependency of consecutive doses of budesonide (800, 1600 and 3200 μg/d) and FP (500, 1000 and 2000 μg/d) were studied. Budesonide increased methacholine PD_20 _from 259 to 467 μg and FP from 271 to 645 μg, both showing a dose-dependency. However, no statistical comparison was made between individual doses. The PD_20 _was increased 1.67-fold and 1.96-fold when the patients were switched from the lowest dose to the highest dose of budesonide and FP, respectively. An apparently dose-dependent decrease in the blood eosinophil count was obtained with budesonide but not with FP treatment [39]. In contrast, no significant differences were observed for either treatment, when morning or evening PEF, symptom scores, and consumption of β_2_-agonist were analysed. Allergen PC_15 _and methacholine PC_20 _values were determined before and after treatment with budesonide at 200, 400 and 800 μg/d for 7 days in a double-blind, randomized and cross-over study (6 day washout period) in eleven atopic subjects with inhalation allergy [40]. The allergen PC_15 _and methacholine PC_20 _were significantly larger for all doses of budesonide as compared with placebo, but there was no significant difference between the 3 doses of budesonide. In an open trial with patients with moderate to severe asthma the effects of progressively increasing doses of budesonide (400, 800, 1600 and 2400 μg/d) were studied [41]. Budesonide decreased the blood eosinophil count in a dose-dependent manner. In a double-blind, randomized placebo-controlled study combining two separate studies, the dose-dependency of the anti-inflammatory effects of budesonide (100, 400 and 1600 μg/d) was assessed in patients with mild asthma (n = 31). Based on trend analysis, there were dose-dependent changes in exhaled NO, sputum eosinophils and PC_20 _to inhaled budesonide but a plateau response of exhaled NO was found at a dose of 400 μg/d [42]. In a study with a novel ICS ciclesonide, its effects were studied in a parallel-group, double-blind, placebo-controlled, randomized cross-over study (washout period 3–8 weeks) in patients (n = 29) with mild to moderate asthma [43]. Compared with placebo, ciclesonide for 14 days (100, 400 and 1600 μg/d) reduced airway responsiveness to AMP by 1.6, 2.0 and 3.4 doubling doses, respectively, and this effect was dose-dependent. A significant reduction in the percentage of eosinophils in induced sputum was observed after 400 and 1600 μg daily ciclesonide, but this was not dose-dependent. Sputum eosinophil cationic protein (ECP) was significantly reduced after 400 μg daily ciclesonide only, and no dose-dependent effect was seen. In a recent single-cohort, prospective placebo-controlled study with four 1 week periods with nonsteroid-treated asthmatic patients (n = 15) the effects of different doses of BDP (100, 400 and 800 μg/d) were measured on FEV_1_, exhaled nitric oxide (FENO) and methacholine PC_20 _[44]. All doses of BDP resulted in a significant change in FEV_1 _and methacholine PC_20 _from baseline or placebo treatment, but with no significant separation of active BDP doses. All doses of BDP resulted in a significant change in FENO from placebo treatment, but with significant separation of only the 100 μg and 800 μg doses by FENO. Another study assessed the dose-response relationship of the anti-inflammatory effects of BDP (50, 100, 200 and 500 μg/d) in the treatment of mild to moderate asthma for 8 weeks in a randomised, placebo-controlled, double-blind trial of parallel-group design [45]. Maintenance ICS therapy was discontinued and patients were randomised to different treatment groups and inflammatory markers such as exhaled NO, sputum eosinophil counts and PD_15 _to saline were followed. There was a significant linear relationship between BDP dose and exhaled NO concentration, FEV_1 _and changes in sputum eosinophils at the end of treatment. In contrast no relationship was found between BDP dose and PD_15 _to saline. However, the results of this study may be confounded because the patients were treated with oral prednisolone for two days in the beginning of the study.

In a recent randomized and double-blinded study, 12 atopic mild stable asthmatic subjects were treated with placebo or mometasone furoate (100, 200 and 800 μg/d) for six days [46] in a cross-over fashion. All three doses of MF demonstrated similar attenuation of early responses and allergen-induced airway hyperresponsiveness relative to placebo with no dose-response relationship. In contrast, the late maximal % fall in FEV_1 _after placebo treatment was 24% and was significantly reduced in a dose-dependent manner to 12%, 11% and 6% for the 100, 200 and 800 μg daily treatments. The allergen-induced sputum eosinophilia (×10^4 ^cells/ml) 24 h after challenge during placebo treatment was 60.2 and was significantly reduced to 24.0, 15.3 and 6.2 for the 100, 200 and 800 μg daily treatments, respectively. Although a statistically significant dose-response relationship was present, the difference between the lowest and highest dose (8-fold difference) for late maximal fall in FEV_1 _or allergen-induced sputum eosinophilia was less than the difference between placebo and the lowest dose of MF.

Taken together, the results suggest that there is tendency towards slightly higher anti-inflammatory efficacy with higher doses of ICS. At the moment there are only a few studies that assess the dose-dependency of the anti-inflammatory effects of ICS. Most of these studies included only small numbers of patients. However, despite the 4–8–16-fold differences in the doses of ICS studied, it has not been easy to demonstrate the dose-dependency of the anti-inflammatory effects of inhaled glucocorticoids. Thus, based on the scarce published evidence we would predict that doubling of the commonly used low to moderate doses of ICS is likely to produce only a small increase in the anti-inflammatory effect, suggesting that inflammation may be suppressed in most patients by relatively low doses of ICS.

### Is there a dose-response with the adverse effects of ICS?

Glucocorticoids suppress corticotrophin levels, which may eventually lead to atrophy of the adrenal cortex and diminished levels of endogenous cortisol. The diminished levels of endogenous cortisol or reduced cortisol excretion have been used as markers of systemic activity of ICS. These systemic effects may include osteoporosis, behavioural effects, growth suppression, posterior subcapsular cataracts, risk for ocular hypertension and glaucoma as well as skin thinning and bruising [47]. In the following sections the literature on the dose-related effects of different steroids on HPA axis as well as on local adverse effects is discussed.

#### Studies included in the systematic review

Of the 14 studies included in this review, in 8 the effects on HPA-axis suppression were analysed. No data on the effects of BDP, budesonide or TAA on HPA-axis were reported. Six of the 7 randomised, double-blind dose-response studies with FP also analysed its effect on HPA axis, measuring either basal morning cortisol levels, post-cosyntropin stimulation test levels or urinary excretion of cortisol metabolites (**Table 2b, **see Additional file 1). Only one study reported a statistically significant dose-response effect (3% decrease per doubling dose of FP) in morning plasma cortisol levels [20] and one study [21] reported slight transient reductions in urinary free cortisol and urinary 17-hydroxy steroids in the group receiving the highest dose of FP (1000 μg/d). However, in 5 studies made with FP, no dose-related effects on HPA-axis suppression were described (**Table 2b, **see Additional file 1). There was no indication for the dose-dependent HPA-axis suppression in 2 studies with mometasone furoate. One needs to note that these studies were not planned and powered to detect differences in systemic or adverse effects.

#### Beclomethasone dipropionate – other literature

The dose-related effects of HFA-BDP (200–800 μg/d) were studied in 43 steroid-naïve asthmatic patients in a randomized double-blind fashion for 14 days [48]. When the HFA-BDP dose increased a greater decrease in the percent change from baseline in steady state 24 h urinary free cortisol was found suggesting a dose-response. Despite the observed statistically significant differences between placebo and the two highest dose-groups in mean percent change in 24 h urinary free cortisol, only one patient among all the treatment groups fell below the reference range for this parameter. In another small, randomized study 26 steroid-naïve asthmatic patients were treated with increasing doses of BDP (400 – 1600 μg/d) [49]. Only the highest dose of BDP produced a significant suppression of 24 h urinary free cortisol. In a recent Cochrane review [10], the dose-response relationship of BDP on HPA axis function was analysed. Only two small studies with adult patients not treated with oral steroids were identified, and showed no effect on morning plasma cortisol by two to five-fold increase in the BDP dose.

#### Budesonide – other studies

A randomized double-blind study with consecutive dose design [39] comparing FP (500–2000 μg/d) and budesonide (800–3200 μg/d) reported that budesonide, but not FP (or at least to a lesser extent) reduced 24 h urine cortisol excretion, plasma-cortisol and serum osteocalcin in a dose-related manner. Similar results have been reported from an open, randomized, parallel group trial with budesonide at doses of 400, 800, 1600 and 2400 μg/d for 2 weeks at each dose level, in adult patients with moderate to severe asthma [41]. Budesonide decreased the 24 h urinary cortisol excretion, serum cortisol and osteocalcin in a dose-dependent manner. In a randomized, double-blind parallel-group study [33], budesonide (1600 μg/d for 12 weeks) induced a mean change from baseline in synthetic corticotrophin (cosyntrophin)-stimulated plasma cortisol levels that was significantly different from placebo and the lowest dose of budesonide. However, the difference from placebo was only 10%, and all other doses of budesonide were not statistically different from placebo. In contrast, the mean basal morning plasma cortisol levels among different budesonide treatment groups and placebo did not differ. In a randomized cross-over study [50], budesonide (1600 μg/d) reduced serum osteocalcin and blood eosinophil count as compared with placebo, but these effects were not dose-dependent. In contrast, budesonide (400–1600 μg/d) had no significant effects on adrenal function as assessed by 8 am serum cortisol or overnight urinary cortisol excretion. In a recent open study, budesonide (400–1600 μg/d) was given to patients with mild to moderate asthma (n = 26) sequentially for 3 weeks each dose, a total of 9 weeks [38]. There was a significant dose-related suppression of morning cortisol levels and overnight urinary cortisol values, but not of serum osteocalcin. For example, the percentages of patients with a stimulated plasma cortisol response less than 500 nM were 7% at baseline, 13% at 400 μg/d, 40% at 800 μg/d and 66% at 1600 μg/d. The authors reported that the proportions of patients with a beneficial airway response together with a minimal systemic response – that is, an optimal therapeutic index – were approximately 50% at all three doses of budesonide. However, the proportion of patients with a good airway response together with a marked systemic response – that is, a suboptimal therapeutic index – increased from 4% at low dose to 38% at high dose [38]. In a recent Cochrane meta-analysis, statistically significant, dose-dependent suppression by budesonide of 24 hour urinary free cortisol excretion and serum cortisol post synthetic ACTH infusion over the dose range 800 – 3200 μg/d were apparent, but the authors concluded that the clinical significance of these findings is unclear [12].

#### Fluticasone propionate – other literature

FP has also been shown to suppress 8 am serum cortisol and urinary cortisol/creatinine ratio in a dose-dependent manner in a single-blind placebo-controlled cross-over study for 9 days in patients (n = 12) with mild to moderate asthma [51]. Similar dose-dependent suppression of adrenocortical activity was reported in four other studies with patients with mild to moderate asthma from the same research group [52-55]. Interestingly, the suppressive effects of FP on adrenocortical activity were greater than those observed on osteocalcin or eosinophils.

A Cochrane review [11] collected data on the effects of FP on HPA-axis function. Significant differences were not apparent between any daily dose of FP in the range of 100–1000 μg/d and placebo on basal plasma cortisol values or urinary cortisol excretion. However, the authors were not able to make a meta-analysis of the cortisol values. In another Cochrane review [32] the same authors found no evidence for dose-dependent suppression of HPA function. However, no decent meta-analysis could be made due to limited availability of data. In contrast to these findings another meta-analysis [47] found that FP exhibits a significantly steeper dose-related systemic bioavailability than BDP, budesonide, or triamcinolone when 21 studies of urinary cortisol levels and 13 studies of suppression of 8 am plasma cortisol levels were analysed. Thus, there clearly exists a discrepancy in the published literature concerning the systemic effects of FP.

Based on the recent Cochrane review and meta-analysis [32] it seems obvious that there is a dose-response relationship in the appearance of local side-effect hoarseness and/or dysphonia so that FP at doses of 400–500 μg/d and 800–1000 μg/d has a significantly higher risk than at lower doses (50–100 μg/d). Similarly FP at doses of 50–100 μg/d induces significantly less oral candidiasis than at doses of 800–1000 μg/d. However, there seemed to be no significant difference in the incidence of sore throat/pharyngitis between any of the FP doses. Another systematic review [16] collected data from fluticasone studies and calculated NNT (number needed to treat) to prevent worsening of asthma and NNH (number needed to harm) to induce oral candidiasis. Three patients needed to be treated with fluticasone 100 μg/d to prevent worsening of asthma (NNT 3), and for fluticasone 1000 μg/d the NNT was 2.1 patients. In contrast, the dose-response curve for side effects was steep. For a dose of fluticasone 100 μg/d, oral candidiasis developed in one of every 90 subjects treated (NNH 90), whereas the NNH for fluticasone 1000 μg and 2000 μg daily were 23 and 6, respectively.

#### Triamcinolone acetonide – other literature

In two randomized studies, TAA in the dose range of 400–1600 μg/d [50,51] did not significantly affect 8 am serum cortisol or the 24 h or overnight urinary excretion of corticosteroid metabolites. In an open non-controlled 6 months study with 400–800–1600 μg/d TAA the plasma cortisol levels before and after cosyntrophin injection were analysed in patients with asthma [56]. Although all treatment regimens caused some reduction in the 24 h excretion of corticosteroid products, none of the mean values was below the normal ranges and no significant suppression in the cosyntrophin test was seen. The mean data indicated that TAA had overall no significant effect on adrenal function at any dose or at any time. However, three patients exhibited some reduction in adrenal function. In another small, randomized study 26 steroid-naïve asthmatic patients were treated with increasing doses of TAA (800 – 3200 μg/d) [49]. Only the highest dose of TAA produced a significant suppression of 24 h urinary free cortisol.

### Conclusions on the effects of ICS on HPA axis and local side effects

Taken together, the data on the systemic adverse effects of ICS is conflicting and seems also to reflect the study design. Several studies have measured only the basal morning cortisol levels or levels after stimulation with high cosyntrophin doses. However, these may be insensitive markers for HPA-axis suppression [47]. Different, a possibly more sensitive endpoint could be plasma cortisol profile during 20–24 h period, which has been shown to be affected by a short course of fluticasone and/or budesonide or even after single inhaled doses [57-59]. There is disagreement between the relative potency of budesonide and FP on HPA-axis function. In addition to the different ways to measure HPA-axis function, this may be due to the use of different inhalers, duration of the treatment period, the selection of the patient group or different design and sponsoring of the studies by pharmaceutical companies. In addition there are differences in the delivery of ICS between normal subjects and patients with asthma and in patients with severe versus mild asthma [60-62]. Although generally safe, it appears that there is at least some degree of dose-dependency in the HPA-axis effects of inhaled steroids. Some smaller studies [39,41,54] suggest that there is a significant decrease in the therapeutic index with higher doses of ICS. Recently, a statistical meta-analysis using regression was performed for parameters of adrenal suppression in 27 studies [47]. Marked adrenal suppression, and thus a marked risk for systemic adverse effects, occurs at doses of ICS above 1500 μg/d (budesonide and BDP) or 750 μg/d (FP), although there is a considerable degree of inter-individual susceptibility. Meta-analysis showed significantly greater potency for dose-related adrenal suppression with FP compared with BDP, budesonide, or TAA. The author concludes that ICS in doses above 1500 μg/d (750 μg/d for FP) may be associated with a significant reduction in bone density [47]. Long-term, high-dose ICS exposure increases the risk for posterior subcapsular cataracts, and to a much lesser degree, the risk for ocular hypertension and glaucoma. Skin bruising, which correlates with the degree of adrenal suppression, is most likely to occur with high-dose exposure [47].

## Adding a long acting-β_2_-agonist (LABA)

### The rationale

LABA provide long-lasting relaxation of airway smooth muscle, while the ICS provide potent topical anti-inflammatory action. In addition to these complementary actions, β_2_-agonists may have several other actions that may contribute to their efficacy in relieving asthma symptoms. β_2_-Agonists inhibit plasma exudation in the airways by acting on β_2_-receptors on postcapillary venule cells. They inhibit the secretion of bronchoconstrictor mediators from airway mast cells and may inhibit release of mediators from eosinophils, macrophages, T-lymphocytes and neutrophils. In addition, β_2_-agonists may have an inhibitory effect on the release of neuropeptides from sensory nerves [63]. Corticosteroids may also increase the expression of β_2_-receptors in inflammatory cells to overcome the desensitisation in response to chronic β_2_-agonist exposure [64]. In addition, LABA may prime the glucocorticoid receptor facilitating activation by corticosteroids [65,66].

### Design of 12 LABA add-on studies included in the review

The literature search identified 3 studies with formoterol [67-69] and 9 studies with salmeterol [70-78]. All these studies included adult or adolescent patients with symptomatic asthma. Generally, patients used low to moderate doses of inhaled glucocorticoids. In two studies [68,73] previous use of ICS was not required. In all studies PEF or FEV_1 _reversibility of at least 10–15% was required (**Table 3, **see Additional file 1). Diurnal or period PEF variation >15% was required in four studies. FEV_1 _of >(40)–50% of predicted and a clearly positive symptom score was required in most studies (**Table 3, **see Additional file 1). In general, the mean FEV_1 _(% predicted) varied between 61 and 87% in different studies, being 61–70% in 4 studies, 70–80% in 3 studies, 81–87% in two studies and was not reported in three studies. The mean absolute PEF values varied from 299 to 404 L/min and FEV_1 _from 2.12 to 2.54 L (**Table 5, **see Additional file 1). Thus, the patient population in these studies represents mainly those with moderate to severe persistent asthma. This as well as the fact that patients with recent exacerbations are excluded may produce a selection bias, compared with the real life. In one study [78] patients were required to have at least two exacerbations during the previous year to be eligible for the inclusion in the study. One study [68] was performed in patients mainly affected with mild persistent asthma. In salmeterol and formoterol studies, the comparison dose of ICS was increased 2–2.5 (-4)-fold, whereas in the formoterol study [67] the comparison dose of budesonide was 4-fold higher (**Table 4, **see Additional file 1). Another significant difference between formoterol and salmeterol studies is that in the formoterol [67] study the main outcome parameter was the incidence of exacerbations whereas the salmeterol studies mainly focused on lung function and asthma symptoms. Most studies allowed a constant dose of theophylline but not oral steroid use (**Table 3, **see Additional file 1). Six out of the 12 studies excluded patients having previous exacerbations (generally during previous month). Only 2 studies lasted one year [67,68], whereas most studies lasted at least 24 weeks. Most reports did not identify whether the study were performed by respiratory specialists or general practitioners. All studies were financially supported by pharmaceutical companies.

### Lung function and asthma symptoms

#### Formoterol – studies included in this systematic review

**Figure 3:**
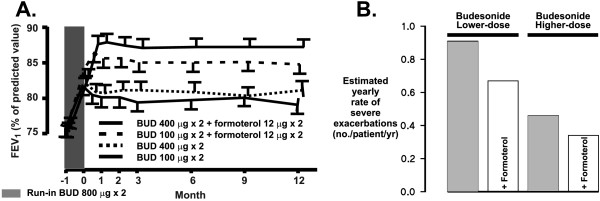
Formoterol add-on study showing forced expiratory volume in one second (FEV_1_) (panel A, from ref 64 with permission) and the estimated yearly rates (no. patients/year) of severe asthma exacerbations in the different treatment groups of the study (panel B). For estimated yearly rate of exacerbations, the P-values given were formoterol *vs *placebo P = 0.01 and lower *vs *higher dose of budesonide P < 0.001.

The addition of formoterol was compared with the increase (4-fold) in the dose of inhaled budesonide (from 200 μg/d to 800 μg/d) in patients with moderate to severe symptomatic chronic asthma [67]. The patients (n = 852) in this study had a FEV_1 _of at least 50% of predicted (mean 75–76%) with an increase in FEV_1 _≥15% after inhalation of terbutaline. Addition of formoterol was superior to the increase in steroid dose in increasing FEV_1 _and morning PEF (Figure 3A; **Table 5, **see Additional file 1). Similarly, addition of formoterol was equal or superior to the 4-fold increase in ICS dose in reducing day- or night-time symptom scores or rescue medication use (**Table 6, **see Additional file 1). Most importantly, the effect of formoterol was sustained over the one-year treatment period. In this study, no statistical comparison was made between the low-dose budesonide + formoterol and high dose budesonide groups.

Another study [69] compared the addition of formoterol (4.5 μg bid) to a small dose of budesonide (160 μg/d) in single inhaler (Symbicort^®^) with an increased dose of budesonide (400 μg/d) in adults with mild to moderate asthma (mean FEV_1 _81–82%) not fully controlled on low doses of ICS alone. The increase in mean morning and evening PEF was significantly higher for budesonide/formoterol compared with budesonide alone. In addition, the percentage of symptom-free days and asthma control days were significantly improved in the budesonide/formoterol group. Budesonide and formoterol decreased the relative risk of an asthma exacerbation by 26% as compared with higher dose budesonide alone.

The results of the formoterol study [67] on the benefits of addition of formoterol were confirmed in patients with mild asthma (mean FEV_1 _86–87% of predicted and using approximately 1 rescue inhalation per day) [68]. In this study, the addition of formoterol was superior to doubling the dose of budesonide in increasing FEV_1 _and morning PEF in the patients already treated with a low dose of ICS, but not in steroid-naïve patients (**Table 5**), or in reducing the percentage of days with symptoms, number of rescue inhalations or nights with awakenings in the patients with mild persistent asthma already treated with low doses of ICS (**Table 6, **see Additional file 1).

A subgroup of the patients participating in the formoterol study [67] was analysed for asthma quality of life parameters using the Asthma Quality of Life Questionnaire (AQLQ) [79]. Following randomisation there was a significant increase in the AQLQ score only in the group with higher budesonide + formoterol group. Although the patterns of mean responses for AQLQ scores and for the clinical variables were very similar, correlations between change in AQLQ scores and change in clinical measures over the randomized period were only weak to moderate (maximum r = 0.51). The data confirm that the benefit from the addition of formoterol is sustained. However, instead of improving pulmonary function parameters patients are usually more interested in how their normal everyday life and activities are limited by the disease. The analysis of AQLQ parameters and their comparison with the clinical data in that analysis also suggest that if only pulmonary function parameters are to be analysed, the benefits of addition of LABA to the treatment may be over-estimated. Also, it should be noted that no correlation has been found between measures of pulmonary function and daytime asthma symptoms [80].

#### Formoterol – other literature

As compared with the abovementioned three studies, similar superiority of addition of formoterol on morning PEF, rescue medication use and asthma symptoms were reported in an open randomised parallel-group study comparing the addition of formoterol to the low-dose BDP with 2-fold higher dose of BDP in patients suffering from symptomatic asthma, despite the use of inhaled BDP [81].

#### Salmeterol – studies included in this systematic review

Addition of salmeterol as compared with the increase in the dose of ICS BDP or FP has been studied in 9 randomised parallel group studies with 3651 patients with moderate to severe persistent asthma (**Tables 3 and 4, **see Additional file 1). Addition of salmeterol improved FEV_1 _better than increasing the dose of ICS 2–4-fold in 5 studies (analysed in 6 studies) and mean morning PEF in 7 studies (analysed in 9 studies), respectively (**Table 5, **see Additional file 1). Similarly, addition of salmeterol was significantly better than the increase in the dose of ICS in increasing the number of days or nights without symptoms or without rescue medication or reducing day- or night-time symptom score as well as daytime or night-time rescue medication use in most studies (**Table 6, **see Additional file 1). However, although addition of salmeterol seems to be superior to increased dose of ICS, a statistically significant difference was not always reached (**Tables 5 and 6, **see Additional file 1) in the single studies when FEV_1_, morning PEF, asthma symptom scores or rescue medication use were analysed. Another feature typical of these studies is that the results favour the addition of salmeterol more at early time points and this difference is reduced as the study proceeds.

#### Salmeterol – other literature

Most of the studies mentioned above, (except ref [72]), have recently been analysed in a meta-analysis [13]. In addition, the published meta-analysis included 1 study (n = 488) that remains unpublished at the present. At baseline these patients (n = 3685, aged ≥12) used BDP 200 – 400 – 1000 μg/d or FP 200 – 500 μg/d. The addition of salmeterol to those doses was compared with increasing the dose of BDP or FP up to 2–2.5-fold. The mean FEV_1 _was <75% in most studies included in the meta-analysis and a reversibility of ≥10–15% in PEF or FEV_1 _after inhalation of short-acting bronchodilator was required for inclusion in all but three studies. In patients receiving salmeterol the morning PEF was 22–27 L/min greater and FEV_1 _was 0.10 – 0.08 L greater after three to six months of treatment, compared to the response to increased steroids. Similarly, the mean percentage of days and nights without symptoms was increased 12–15% and 5%, respectively, as well as the mean percentage of days and nights without need for rescue treatment increased 17–20% and 8–9%, respectively.

### Effect of LABA on asthmatic inflammation

The results of the above mentioned studies favour the addition of a LABA instead of increasing the dose of ICS in patients not adequately controlled with low to moderate doses of ICS. However, there have been concerns that regular use of inhaled β_2_-agonists may mask an increase in the underlying airway inflammation in asthma. Also, some proinflammatory effects have been described for β_2_-agonists such as delay of constitutive eosinophil apoptosis [82] or reversal of corticosteroid-induced apoptosis [83]. Furthermore, development of tolerance to their protective effects against various asthma-provoking stimuli has been reported. There is some disagreement whether the addition of formoterol or salmeterol changes the level of pulmonary inflammation in patients already treated with inhaled glucocorticoids or whether they may even mask the inflammation. Three studies [84-86] do not indicate any significant increase in the inflammatory indices following addition of formoterol or salmeterol, whereas treatment of asthma with salmeterol with concomitant steroid tapering has been shown to increase the numbers of eosinophils in sputum [87].

#### Formoterol – studies included in this systematic review

In a randomised, double-blind and parallel-group study (n = 61) with similar inclusion and exclusion criteria than in the formoterol add-on study [67], the effect of adding formoterol (12 μg bid) to a low dose of budesonide (200 μg/d) was compared with a higher dose of budesonide (800 μg/d) for 1 year after a run-in with budesonide (1600 μg/d) for 4-wk [84]. Budesonide (1600 μg/d) during run-in significantly reduced median sputum eosinophils. No significant changes in the proportion of eosinophils, other inflammatory cells, or ECP levels in sputum were observed over the ensuing one year treatment with formoterol + budesonide (200 μg/d) or higher dose budesonide (800 μg/d). Clinical asthma control was not significantly different between both groups.

#### Salmeterol – other literature

In a small study (n = 9) with asthma patients using regular inhaled glucocorticoids and inhaled salbutamol for symptom relief, the addition of salmeterol for 8 weeks was studied in a double-blind crossover placebo-controlled protocol [86]. Bronchoalveolar lavage (BAL) cell profile, albumin and tryptase levels, percentages of CD4^+ ^and CD8^+ ^lymphocytes and lymphocyte activation as assessed as proportions of lymphocytes expressing HLA-DR were measured in BAL samples before and after treatment. There were no significant changes after salmeterol treatment. In another double-blind, parallel-group, placebo-controlled study [85] the effect of addition of salmeterol (50 μg bd) or fluticasone (200 μg/d) for 12 weeks was studied in 45 symptomatic patients with asthma who were receiving ICS (range 100–500 μg/d). Bronchial biopsies and BAL were analysed before and after the treatment. After treatment with salmeterol there was no deterioration of airway inflammation, as assessed by mast cell, lymphocyte, or macrophage numbers in BAL or biopsies, but a significant fall in EG1-positive eosinophils in the lamina propria was found, which was not seen after treatment with FP. The only cellular effect of added FP was a decrease in BAL lymphocyte activation as assessed as proportions of lymphocytes expressing HLA-DR. There was a concurrent improvement in clinical status, more marked with salmeterol than with increased ICS. These two studies thus suggest that adding salmeterol to ICS is not associated with increased airway inflammation. In another study in 13 asthmatic individuals requiring ≥1500 μg ICS daily, the steroid sparing and "masking" effects of salmeterol versus placebo were studied in a randomised, placebo-controlled, double-blind and crossover trial [87]. Subjects were re-stabilised on their original dose of ICS for 4 wk before crossover to the alternative treatment. Corticosteroid doses were reduced weekly until criteria were met for an exacerbation or the corticosteroid was fully withdrawn. Mean ICS dose was reduced significantly more (87%) during salmeterol treatment, than with placebo (69%). Sputum eosinophils increased before exacerbation, despite stable symptoms, FEV_1 _and PEF. In the week before clinical exacerbation, sputum eosinophil counts were higher in the salmeterol-treatment arm as compared with placebo, whereas there were no differences in PC_20 _or serum ECP. Five subjects showed >10% sputum eosinophilia before exacerbation during salmeterol treatment, compared to two receiving placebo. This suggests that the use of salmeterol allowed subjects to tolerate a greater degree of inflammation without increased symptoms or reduced lung function. Thus, during progressive reduction of ICS the bronchodilator and symptom-relieving effects of salmeterol may mask increasing inflammation and delay awareness of worsening asthma. These findings strengthen guideline recommendations that LABA should not be described as sole anti-asthma medication and that they should be used as "add-on" therapy rather than for steroid tapering purposes.

The effect of addition of salmeterol (50 μg bd), FP (200 μg/d) or placebo for 3 months on airway wall vascular remodelling has been studied in 45 symptomatic patients with asthma who were receiving treatment with ICS (range 400–1000 μg/d) [88]. Bronchial biopsies were analysed before and after treatment. There was a decrease in the density of vessels of lamina propria after treatment only in the salmeterol group compared to baseline. There was no significant change within the FP or placebo groups and no treatment was associated with increased airway wall vascularity.

### Asthma exacerbations

If there were a marked masking of pulmonary inflammation by LABA, one would expect to see an increase in the number and severity of asthma exacerbations during their long-term use. There is some difficulty in comparing the different studies done with formoterol and salmeterol as the definition of exacerbation varies. In formoterol studies [67,68] a severe exacerbation was defined as need for treatment with oral corticosteroids, as judged by the investigator, or hospital admission or emergency treatment for worsening of asthma or a decrease in morning PEF >25%–30% from baseline on two consecutive days. In contrast, in the salmeterol "add-on" studies the exacerbation was not defined at all or was more loosely defined for example as "a clinical exacerbation", "any worsening of asthma symptoms requiring a change in prescribed therapy, other than increased use of rescue medication" or "any asthma event that required treatment with oral or parenteral steroids".

#### Formoterol – studies included in this systematic review

**Figure 4:**
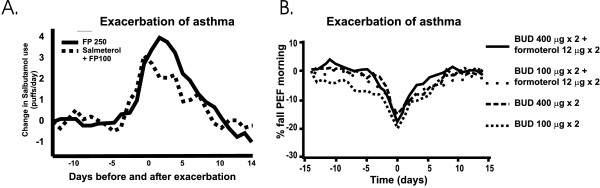
A. Change in supplemental salbutamol use before and after exacerbation in patients treated with fluticasone and salmeterol combination or with high-dose fluticasone (with permission from ref 90), B. Change in morning PEF (percent fall from day -14) over the 14 d before and 14 d after an exacerbation in relation to treatment as analyzed from a subgroup of a FACET study (with permission from ref 89).

In the formoterol study [67] the main outcome parameter was the rate of exacerbations during combination therapy. The results show that the 4-fold increase in the dose of budesonide reduced the rates of severe and mild exacerbations by 49% and 37%, respectively, whereas addition of formoterol to the lower dose of budesonide reduced the rates of severe and mild exacerbations by 26% and 40%, respectively. Patients treated with formoterol and the higher dose of budesonide had the greatest reductions, 63% and 62%, respectively (Figure 3B; **Table 7, **see Additional file 1). This suggests that if frequent asthma exacerbations are a major problem, increasing the dose of ICS may help to reduce the number of exacerbations. The results of the formoterol study [67] as well as the salmeterol meta-analysis [13] suggest that addition of LABA has divergent effects on asthma control: it is superior to the increased steroid dose in improving lung function, but is equal or less efficient in reducing exacerbations (Figure 3AB). The data also suggest that to achieve a better control of asthma exacerbations, the dose of ICS should be increased 4-fold. When 425 exacerbations of the formoterol study [67] were analysed [89], the use of higher dose of ICS or the use of formoterol was shown not to affect the pattern of change in PEF values or in symptoms during asthma exacerbation (Figure 4B).

In contrast to that described in moderate to severe asthma, in the other formoterol study [68] addition of formoterol (6 μg bid) to either the lower (200 μg/d) or higher (400 μg/d) dose of budesonide in patients suffering from mainly mild asthma reduced the risk of the first asthma exacerbation by 43% (RR = 0.57, 95% CI 0.46–0.72). There was also a significant 52% reduction in the rate of severe exacerbations (RR = 0.48; 95% CI 0.39–0.59). In addition, significant improvement was observed for the rate of severe exacerbations (RR = 0.58, 95% CI 0.44–0.76). Thus, the data suggest that there may be a difference in the effect of ICS and formoterol on the exacerbations between mild and moderate to severe asthma so that in mild asthma addition of LABA may be more efficient in preventing exacerbations, whereas in moderate to severe asthma increasing the dose of ICS may be more efficient (**Table 7, **see Additional file 1). However, the formoterol studies [67,68] are not fully comparable in that way that in the other study [67] the increase in the dose of budesonide was 4-fold whereas in the other study [68] it was 2-fold.

Another study [69] compared the addition of formoterol (4.5 mg/d) to a small dose of budesonide (160 μg/d) in single inhaler (Symbicort^®^) with an increased dose of budesonide (400 μg/d) in adults with mild to moderate asthma (mean FEV_1 _81–82%) not fully controlled on low doses of ICS alone. Budesonide/formoterol combination significantly decreased the relative risk of an asthma exacerbation by 26% as compared with higher dose budesonide alone. In contrast, the estimated risk of having a severe exacerbation was 6% lower in patients treated with budesonide/formoterol compared with those receiving budesonide alone, but this was not statistically significant.

#### Salmeterol – studies included in this systematic review

Only two studies [70,78] of those included in this systematic review reported the actual monthly or annual rates for moderate or severe exacerbations. In those studies there were no significant differences in the yearly rate of exacerbations or percentages of patients experiencing at least exacerbation. The other studies generally reported the percentages of patients experiencing at least one exacerbation (Table 7). In salmeterol studies, the data were presented mostly in a form, which did not allow us to calculate the yearly rate of exacerbations.

#### Salmeterol – other literature

In the salmeterol studies lasting 3–6 months the numbers of patients with exacerbations were analysed. The meta-analysis [13] revealed that fewer patients experienced any exacerbation with salmeterol (difference 2.7%), and the proportion of patients with moderate or severe exacerbations was also lower (difference 2.4%). Thus, to prevent one exacerbation 37–41 patients should be treated with salmeterol instead of increasing the dose of ICS. Rather than indicating salmeterol being superior, the result suggests that there is no increased risk for exacerbations with the use of salmeterol. Unfortunately, in most salmeterol studies the severity and/or yearly incidence of exacerbations was not analysed. As one patient can experience more than one asthma exacerbation during the study, the parameter used in the salmeterol studies (proportion of patients experiencing an exacerbation) may not reflect the actual number of exacerbations. Another factor that may affect our interpretation of the effect of these therapies on asthma exacerbations is that in 6 of the 12 LABA studies, patients could be withdrawn from the study if they experienced >1–5 exacerbations (**Table 7, **see Additional file 1). This may underestimate the total incidence of exacerbations, as those patients experiencing several exacerbations were excluded from analysis. However, these are the patients the "add-on" therapies are most frequently prescribed.

Recently, the exacerbation rates and clinical measures of asthma worsening were assessed in an analysis combining results from two double-blind studies (n = 925) comparing addition of salmeterol to low-dose-FP with increasing the dose of FP 2.5-fold [90]. The addition of salmeterol resulted in a significantly lower rate (0.23 vs. 0.39 per patient per year) of exacerbations compared with higher dose FP. Salmeterol combined with low-dose FP was significantly more protective than 2.5-fold higher dose of FP in preventing asthma exacerbations, as assessed by the time to first exacerbation. In both groups clinical indicators of worsening of asthma showed parallel changes before asthma exacerbation, and greater improvements in morning PEF, supplemental salbutamol use and asthma symptom score were observed after exacerbation with salmeterol compared with higher dose FP (Figure 4A). Thus, the ability to detect deteriorating asthma and the severity of exacerbation is not negatively affected by salmeterol.

### Adverse effects of LABA

The addition of LABA to the treatment regimen usually results in a slight increase in those pharmacologically predictable adverse events such as tremor and tachycardia. However, generally these do not lead to the discontinuation of the treatment. In the formoterol studies [67-69], no significant differences were reported on the adverse effects between the groups, but no detailed data was presented. Also, in the salmeterol studies [70-78], the incidence of adverse events was very low and generally was not different between the treatment groups. Although LABA appear to be generally very safe, one should not forget that they are generally not suitable for patients with symptomatic coronary heart disease or hyperthyroidism and may provoke more severe adverse events such as supraventricular tachycardias, atrial fibrillation and extrasystoles. Rarely hypersensitivity reactions and painful muscular cramps may occur. Also one should note that the "add-on" studies included in this review are not originally planned and powered to detect significant differences in the adverse effects.

## Adding a leukotriene receptor antagonist (LTRA)

### Rationale

Cysteinyl leukotriene receptor-antagonists (LTRA), such as montelukast, pranlukast and zafirlukast, are a new class of asthma medication, whose role in the stepwise management of asthma has not yet been fully established. Leukotriene antagonists blunt the obstructive response and have weak anti-inflammatory activity. In some studies corticosteroids are not very effective inhibitors of cysteinyl leukotriene pathways, at least when assessed by their inability to reduce cysteinyl leukotriene concentrations [91,92] and thus combination of these therapeutic classes may offer some benefit.

#### Montelukast – studies included in this systematic review

**Figure 5:**
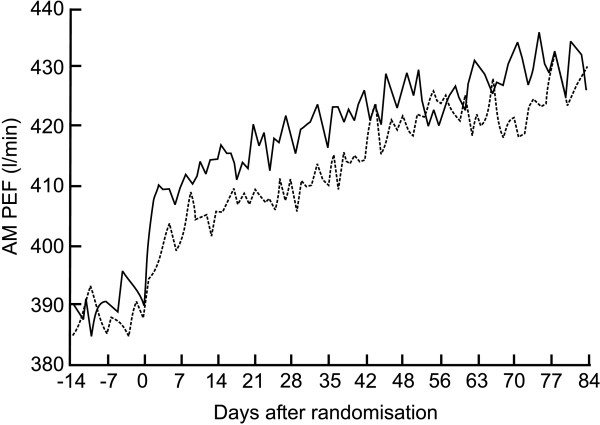
Effect of addition of montelukast (10 mg/d) or doubling the dose of ICS on morning peak expiratory flow (AM PEF) over 12 week treatment period in patients not adequately controlled by budesonide 800 μg/d (solid line = montelukast + budesonide 800 mg/d, dashed line = budesonide 1600 μg/d) (with permission from ref 93).

We identified one randomised, double-blind, parallel-group 16 week study (Jadad score 3) comparing the addition of montelukast (10 mg/d) to budesonide (800 μg/d) with doubling the dose of budesonide (1600 μg/d) in patients inadequately controlled on inhaled budesonide (800 μg/d, n = 448) [93]. The inclusion criteria were: patients (aged 15–75 years) who were not optimally controlled as judged by the investigators in spite of a regular ICS (600–1200 μg/d for BDP, budesonide, TAA, flunisolide or 300–800 μg/d for FP). Patients were required to have FEV_1 _≥50% predicted at visits 1 and 3, with a ≥12% bronchodilator response and symptoms requiring β-agonist treatment of at least 1 puff/day during the last 2 weeks of the run in period (total 4 weeks). Both groups showed progressive improvement in several measures of asthma control compared with baseline. Mean morning PEF improved similarly in the last 10 weeks of treatment compared with baseline in both the montelukast + budesonide group and in the double dose budesonide group (33.5 *vs *30.1 L/min). The improvement in montelukast + budesonide group was faster as the mean morning PEF was significantly higher during days 1–3 after start of treatment in this group as compared with the double dose budesonide group (20.1 *vs *9.6 L/min) (Figure 5). Both groups showed similar improvements with respect to rescue β_2_-agonist use, mean daytime symptom score, nocturnal awakenings, exacerbations, asthma free days, peripheral blood eosinophil counts, and asthma specific quality of life. The authors conclude that addition of montelukast to ICS offers comparable asthma control to doubling the dose of ICS. However, it needs to be remembered that, in most cases, to obtain a statistically significant improvement in asthma control at least a 4-fold increase in the dose of ICS is needed (see above).

#### Montelukast – other literature

A large (n = 639) study [94] recruited patients with asthma not optimally controlled by ICS (stable dose equivalent to budesonide 400–1600 μg/d). The patients were required to have FEV_1 _≥55%, a bronchodilator response greater than 12%, symptoms and rescue β_2 _agonist use of at least 1 puff/day. The mean FEV_1 _at baseline was 81% predicted. The patients were randomised to obtain either montelukast (10 mg/d) or placebo in a double-blind manner. The ICS dose remained constant throughout the study. The primary efficacy end point was the percentage of asthma exacerbation days. The major advantage of this study is that this study adopted several different definitions for asthma exacerbation days from previously published other studies, making comparison to other studies more easy. The median percentage of asthma exacerbation days was 35% lower (3.1% *vs *4.8%, p = 0.03) and the median percentage of asthma free days was 56% higher (66.1% *vs *42.3%, p = 0.001) in the montelukast group than in the placebo group. Thus, the NNT with montelukast to avoid one exacerbation day was 13, and the NNT to avoid one day not free of asthma – that is, to gain an asthma free day – was 10. Patients receiving concomitant treatment with montelukast had significantly less (25.6% *vs *32.2%, p = 0.01) nocturnal awakenings, and significantly greater reductions in β_2_-agonist use (17.26% v 4.92%, p = 0.05, baseline use was 3.2–3.3 puffs/day), and morning PEF (16.86 L/min *vs *11.30 L/min, p = 0.05, baseline 365–373 L/min). No significant difference was found in asthma specific quality of life or in morning FEV_1_. The results of this study suggest that although the effect of montelukast on endpoints such as morning PEF, FEV_1 _and rescue β_2_-agonist use are only small or modest, addition of montelukast may produce a significant improvement of asthma control by reducing the number of asthma exacerbation days.

In another study with patients (n = 642) with symptomatic persistent asthma despite the treatment with BDP (400 μg/d), addition of montelukast (10 mg/d), improved morning FEV_1 _and PEF, asthma symptom score and the percentage of asthma exacerbation free days better than placebo during 16 week treatment period [95]. The increase in morning FEV_1 _was approximately 140 mL and in morning PEF 10 L/min. There was a tendency towards reduced rescue medication use with the combination therapy, but the reduction was only 0.2 puffs/day. Addition of montelukast to ICS seemed to prevent the increase in the number of peripheral blood eosinophils seen in other treatment groups.

In an atypical "add-on" study (randomised double-blind, placebo-controlled and crossover trial), addition of montelukast (10 mg/d) was compared with placebo in patients with asthma (n = 72) and symptoms despite treatment with ICS and additional therapy [96]. Most of the patients used several different types of combination therapy, except leukotriene antagonists, at baseline. The inclusion criteria were defined as "any patient with physician diagnosis of asthma in whom the recruiting physician felt a trial of montelukast was indicated for continued asthma symptoms despite other anti-asthma therapy". A current worsening of asthma requiring oral corticosteroid treatment, or worsening in the preceding month were both exclusion criteria, but did not exclude any of those referred for inclusion in the trial. In this setting corresponding to a typical hospital outpatient clinic, addition of montelukast did not result in any significant change in symptom scores, rescue inhaled β_2_-agonist use, or morning or evening PEF. When treatment response was defined as a 15% or greater increase in mean PEF recordings, there were four responders to montelukast and seven responders to placebo. Although several points in this study may be criticised (loose inclusion criteria, small sample size, short 2 week treatment period, no wash-out period, encapsulation of the tablets, exacerbations not analysed as end-point), the results suggest that the effects of montelukast are not as evident in unselected population than in the more clearly defined patients included in other trials [93-95].

The additional anti-inflammatory activity obtained by adding montelukast to the treatment regimen has been assessed in three randomised, double-blind, cross-over studies lasting 10 days–8 weeks. In one study [97], addition of montelukast (10 mg/d) to salmeterol (50 μg bid) and fluticasone (250 μg bid) combination was compared with placebo in patients with mild-moderate asthma for 3 weeks. Compared with salmeterol/fluticasone run-in period, adding montelukast was better (p < 0.05) than placebo for inflammatory markers such as AMP-threshold, recovery, exhaled NO, and blood eosinophils but not for lung function. In another study [98], addition of montelukast for 8 weeks to FP (100 μg bid) was compared with placebo in patients with mild asthma. There were no differences in FEV_1 _or histamine PC_20 _between the two treatment regimens. There was no difference in the efficacy of either treatment in decreasing T cell, CD45RO+, mast cell or activated eosinophil numbers in bronchial biopsies. In a third study [99], the addition of montelukast (10 mg/d) to budesonide (400 μg/d) for 10 days to steroid-naïve patients with asthma was reported not to produce any additional anti-inflammatory benefit when compared with budesonide alone in reducing airway hyperresponsiveness or sputum eosinophilia.

#### Zafirlukast – other studies

Addition of high-dose zafirlukast (80 mg b.i.d.: 4-fold greater than the approved dose) improved asthma control better than placebo in patients (n = 368) on high-dose ICS (1000 – 4000 μg/d) [100]. Compared with placebo, addition of zafirlukast improved morning and evening PEF and reduced daytime symptom score and rescue medication use [100]. According to a recent meta-analysis [101,102], in symptomatic asthmatic adults, addition of zafirlukast (80 mg bid) to ICS did not reduce the risk of an exacerbation requiring systemic steroids after 12 weeks of treatment, compared to double dose ICS [RR = 1.08; 95% CI 0.47, 2.50]. There were no differences in any other measure of outcome. Higher doses of zafirlukast than currently licensed were associated with increased risk of liver enzyme elevation.

### Conclusions on adding a LTRA

According to recent meta-analyses (12 adult studies and 1 in children) [101,102], leukotriene antagonists (zafirlukast or pranlukast at 2–4 times the licensed dose) combined with ICS (300–2000 μg/d BDP equivalent) reduce the number of patients with exacerbations that require systemic corticosteroids, compared to ICS alone [RR = 0.34; 95% CI 0.13, 0.88]. This equates to 20 patients (95% CI 1,100) treated to prevent one needing systemic corticosteroids. There was no difference in side effects [101,102]. The addition of licensed doses of LTRA to ICS resulted in a non-significant reduction in the risk of exacerbations requiring systemic steroids (two trials, RR 0.61, 95% CI 0.36, 1.05). This systematic review did not include the recent study comparing the addition of montelukast to double-dose ICS [93]. As that systematic review did not include any data of LTRA drugs at currently licensed doses compared with high dose ICS, the author came to a conclusion that the addition of LTRA to ICS may modestly improve asthma control compared with ICS alone but this strategy cannot be recommended as a substitute for increasing the dose of ICS [101]. However, based on one relatively large trial [93], the evidence suggests that addition of montelukast may be equal to doubling the dose of ICS. However, one might criticise this conclusion as this study [93] lacked placebo arm, ie. it is possible that increasing (doubling) the dose of ICS does not produce any real improvement in asthma control as compared with lower ICS dose and thus the result showing non-inferiority to double dose ICS might mean no effect at all. Thus, more data is needed to compare the efficacy of LTRA at currently licensed doses with increasing the dose of ICS.

## Adding theophylline

### Rationale

Although theophylline has traditionally been classified as a bronchodilator, its ability to control chronic asthma is greater than can be explained by its relatively small degree of bronchodilator activity. In fact, theophylline has immunomodulatory, anti-inflammatory and bronchoprotective effects that may contribute to its efficacy as an anti-asthma drug [103]. There is some evidence that addition of theophylline to ICS treatment improves pulmonary function and asthma symptoms [104], although all studies have not been able to confirm this result [105].

#### Theophylline – studies included in this systematic review

The addition of theophylline has been compared with doubling the dose of ICS (BDP and budesonide; 400 μg/d → 800 μg/d) in two separate studies with 195 patients with symptomatic asthma for 6 to 12 weeks [106,107]. Theophylline was used at relatively low doses, the mean serum theophylline concentrations were 8.7 and 10.1 mg/L in these studies.

**Figure 6:**
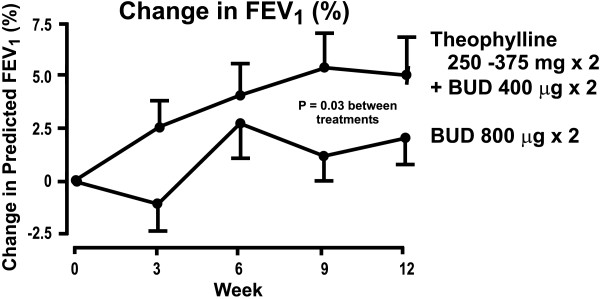
Mean (+- SE) change in FEV_1 _in 31 asthma patients treated with high-dose budesonide (1600 μg/d) and 31 patients given low-dose budesonide (800 μg/d) and theophylline (with permission from ref 106).

In the study (Jadad score 4) of Evans and coworkers [106] addition of low-dose theophylline to budesonide (400 μg/d) was compared with doubling the dose of budesonide (800 μg/d) in a randomised double-blind trial for 3 months. Patients (n = 62) were required to have FEV_1 _predicted normal ≥50%, bronchodilator response of at least 15% and to have symptoms despite the use of ICS (equivalent to budesonide dose of 800–1000 μg/d). The overall treatment effect of addition of theophylline was superior to double-dose budesonide in improving FVC and FEV_1 _(Figure 6), although at single timepoints there were no significant differences between the treatments. There was no significant difference between the treatments in improving home PEF recordings or reducing β_2_-agonist use or symptom scores. There was no difference in the occurrence of possibly drug-related adverse effects between the groups. The statistical power of this study was calculated to detect significant changes over baseline, but not to detect differences (superiority) or non-inferiority between the treatments.

A randomised, double-blind parallel-group study (Jadad score 3) by Ukena and coworkers [107] compared the addition of theophylline to low dose BDP (400 μg/d) with double-dose BDP (800 μg/d) for 6 weeks. Patients (n = 133) were required to have FEV_1 _50–85% predicted normal and a documented reversibility of at least 15% of FEV_1 _over baseline and to be not controlled by BDP (400 μg/d) or equivalent. The sample size of this study was powered to detect equivalence. No significant differences were found between the high-dose BDP and low-dose BDP plus theophylline groups in outcomes such as morning or evening PEF, PEF variability, FEV_1_, daytime or nighttime symptom scores or rescue medication use. Both treatments were well tolerated.

Lim *et al*. [108] recruited asthmatic patients that were symptomatic while being treated with low dose inhaled steroids (400 μg BDP, 200 μg FP or 400 μg BDP daily). Patients (n = 155) were required to have PEF ≥50% of the predicted normal with at least 15% variability in PEF. The patients were randomised to treatment either with low dose BDP (400 μg/d) alone, theophylline plus BDP (400 μg/d) or high-dose BDP (1000 μg/d) for six months in a double-blind trial (Jadad score 5). No significant differences were found between any of the treatment groups in morning PEF, evening PEF, PEF variability, rescue β_2_-agonist use, symptom scores or in the number of exacerbations. Of note is that there were no difference between the low dose BDP alone and high dose BDP groups in any of the parameters. This study was powered to detect superiority of theophylline plus BDP as compared with high-dose BDP. There were no significant differences between the treatment groups for any of the commonly reported adverse effects. The results of this study suggest that when the benefit of an "add-on" therapy is evaluated as compared with double-dose inhaled steroid, additional group using low-dose steroid alone should be included to see whether even the doubling of the dose of steroid produces any benefit to the patient.

### Conclusions on the addition of theophylline

Taken together, the results from two relatively small studies suggest that addition of low-dose theophylline may be equal to doubling the dose of ICS in the treatment of asthma not adequately controlled by low dose of ICS. However, one needs to remember that the effect of doubling the dose of ICS on asthma control is generally small or negligible (see above). Furthermore, a placebo group should be included in these studies to see whether an improvement in asthma control is obtained by doubling the dose of ICS. Thus, more data is needed to confirm the present results. Use of theophylline at concentrations at the lower limit or slightly below the recommended therapeutic range may help to limit the adverse effects.

## Comparison between LTRA, theophylline and LABA as add-on options

### Montelukast versus salmeterol – studies included in this systematic review

Combination of fluticasone (100 μg bid) and salmeterol (50 μg bid) in a single inhaler has recently been shown to provide more effective asthma control than montelukast (10 mg daily) combined with FP (100 μg bid) in a 12 weeks study (randomised, double-blind, double-dummy, Jadad score 3) in patients (n = 447) whose symptoms were suboptimally controlled by ICS only [109]. The inclusion criteria were FEV_1 _between 50% and 80% predicted normal, and at least 1 additional sign of inadequate asthma control during the 7 preceding days. Salmeterol/FP combination was superior to montelukast/FP in improving morning PEF (24.9 *vs *13.0 L/min), evening PEF (18.9 *vs *9.6 L/min), FEV_1 _(0.34 *vs *0.20 L) and shortness of breath symptom score (-0.56 *vs *-0.40) as well as increasing the percentage of days without rescue medication (26.3 *vs *19.1%). In contrast, there was no significant difference in outcomes such as chest tightness, wheeze and overall symptom scores. Asthma exacerbation rates were significantly (P = 0.031) lower in the FP + salmeterol group (2%) than in the FP+ montelukast group (6%). Adverse event profiles were reported to be similar.

A similar study [110] comparing the efficacy of combination of FP (100 μg bid) and salmeterol (50 μg bid) in a single inhaler with combination of montelukast (10 mg daily) and FP (100 μg bid) in a 12 weeks study (randomised, double-blind, double-dummy, Jadad score 4) in patients (n = 725) whose symptoms were suboptimally controlled by ICS (BDP, budesonide, flunisolide 400–1000 μg/d or FP 200–500 μg/day) only. The inclusion criteria were FEV_1 _above 50% and at least 15% bronchodilator response, and asthma symptoms at least at 4/7 days during run-in. Salmeterol/FP combination was superior to montelukast/FP in improving morning PEF (36 *vs *19 L/min), evening PEF (29 *vs *14 L/min), FEV_1 _(0.26 *vs *0.17 L), percentage of symptom-free days (42.9 *vs *31.5%), percentage of symptom-free nights (46.5 *vs *41.1%) as well as increasing the percentage of days without rescue medication (47.9 *vs *46%). In contrast, there was no significant difference in percentage of rescue free nights. The number of patients experiencing at least one asthma exacerbation (any severity) was significantly (P < 0.05) lower in the FP + salmeterol group (9.6%) than in the FP+ montelukast group (14.6%). The percentage of patients who had at least one asthma exacerbation of either moderate or severe intensity was 4.8% in the salmeterol + FP group and 8.4% in the montelukast + FP group, but this difference did not reach statistical significance. The time to the first exacerbation was significantly (P < 0.05) longer in the salmeterol + FP group than in the montelukast + FP group. Adverse event profiles were reported to be similar.

Another very similar study [111] was designed to demonstrate the non-inferiority of combination of montelukast (10 mg daily) and FP (100 μg bid in dry powder inhaler) as compared with combination of FP (100 μg bid in dry powder inhaler) and salmeterol (50 μg bid; metered dose inhaler) on asthma exacerbations. This 48 weeks study (randomised, double-blind, double-dummy, Jadad score 5) included patients (n = 1490) whose symptoms were suboptimally controlled by ICS (equivalent to BDP 200–1000 μg/d). The inclusion criteria were FEV_1 _50–90% predicted and at least 12% bronchodilator response, short-acting β_2_-agonist use of one puff/day or more and asthma symptoms. Salmeterol/FP combination was superior to montelukast/FP in improving morning PEF (34.6 *vs *17.7 L/min), FEV_1 _(0.19 *vs *0.11 L). In contrast, there was no significant difference in nocturnal awakenings and asthma specific quality of life score. The percentage of patients experiencing at least one asthma exacerbation (any severity) was shown to be similar in the FP + salmeterol group (19.1%) than in the FP+ montelukast group (20.1%). Also there was no difference in the time to the first exacerbation between the salmeterol + FP and the montelukast + FP groups. Peripheral blood eosinophils were reported to be reduced significantly more in the montelukast + FP group (-0.04 × 10^3^/μl) than in the salmeterol + FP group (-0.01 × 10^3^/μl). Interestingly more serious adverse events were reported in the salmeterol + FP group.

In another randomised, double-blind, double-dummy, parallel-group study (Jadad score 3) in patients (n = 948) with symptomatic asthma despite treatment with ICS, addition of montelukast (10 mg daily) was compared with addition of salmeterol (50 μg bid) for 12 weeks [112]. Patients were required to have symptoms despite the constant dose of ICS (any brand at any dose) and FEV_1 _between 50% and 80% predicted and at least 12% bronchodilator response. Treatment with salmeterol resulted in significantly greater improvements from baseline compared with montelukast for most efficacy measurements, including morning PEF (35.0 *vs *21.7 L/min), percentage of symptom-free days (24 v 16%) and percentage of rescue-free days (27 *vs *20%). Also total supplemental salbutamol use (-1.90 *vs *-1.66 puffs per day) and nighttime awakenings per week (-1.42 *vs *-1.32) decreased significantly more with salmeterol than with montelukast. Six percent of patients in the salmeterol group experienced a total of 27 asthma exacerbations compared with 5% of patients in the montelukast group who experienced 24 asthma exacerbations during the 12 weeks treatment period. However, the patients experiencing an asthma exacerbation were withdrawn from the study. Thus, annualised incidences of exacerbations cannot be compared [112]. The safety profiles of the two treatments were reported to be similar.

Taken together, addition of salmeterol seems to produce better improvement of asthma control when lung function is assessed than addition of montelukast in patients with asthma suboptimally controlled by small to moderate doses of ICS. However, in one long-term study [111] addition of montelukast to fluticasone was shown to be non-inferior to addition of salmeterol when the percentage of patients with at least one asthma exacerbation was used as the primary endpoint. Whereas addition of salmeterol may produce a better improvement in lung function, addition of montelukast may provide additional anti-inflammatory efficacy to ICS that is reflected in a long-term efficacy on asthma exacerbations. A factor that may produce a selection bias in these studies [109-111] is that a positive response to bronchodilator was required for inclusion. In fact, the reported mean improvements in FEV_1 _in response to β_2_-agonist were 23–24% [109], 27.0–27.4% [110] and 18.4–18.8% [111] in the single studies. This may produce a selection bias favouring long-acting β_2_-agonist. However, one needs to remember that many of those studies done with leukotriene receptor antagonist to prove their efficacy in the treatment of asthma have been performed with patients displaying a significant response to β_2_-agonist. Another factor that might be considered to produce bias is that all the above three studies that report salmeterol to be better have been sponsored by the producer of salmeterol and that study reporting the non-inferiority of montelukast as compared with salmeterol has been sponsored by producer of montelukast.

### Montelukast versus salmeterol – other literature

In addition to the normal clinical endpoints, the effects of addition of salmeterol (50 μg bid) or montelukast (10 mg/d) to the treatment regimen were analysed on AMP bronchial challenge, blood eosinophil counts and exhaled NO in a placebo-controlled, double-dummy, crossover study in patients (n = 20) with persistent asthma not controlled with ICS [113]. For the provocative concentration of AMP causing a 20% fall in FEV_1_, compared to placebo, there were significant differences with the first and last doses of montelukast as well as the first but not the last dose of salmeterol, thus indicating the development of some tolerance with salmeterol. Only montelukast produced a significant, albeit trivial, suppression of blood eosinophil count. There were significant improvements with the first doses of salmeterol for all parameters of lung function. After 2 weeks of treatment, there were significant improvements with both drugs on rescue bronchodilator requirement and morning PEF. There were no significant differences between drugs for any endpoints except blood eosinophils. Thus, the results suggest some anti-inflammatory activity for montelukast when used as an "add-on" therapy.

### Salmeterol versus zafirlukast – studies included in this systematic review

In a randomised, double-blind, double-dummy parallel-group trial (Jadad score 3) addition of zafirlukast (20 mg bid) was compared with the addition of salmeterol (50 μg bid via MDI) for 4 weeks in adult and adolescent patients (n = 429) with persistent asthma [114]. Patients were required to have FEV_1 _percentage predicted normal between 50 and 70% with or without asthma symptoms, or FEV_1 _of 70.1% to 80% of predicted normal values and symptoms or requirement for rescue β_2_-agonist use ≥4 puffs/day or diurnal PEF-variation of more than 20% at two days during 6 days run-in. Both inhaled salmeterol and oral zafirlukast resulted in within-group improvements from baseline in measures of pulmonary function (morning and evening PEF and FEV_1_), asthma symptoms, and supplemental salbutamol use. Salmeterol treatment resulted in significantly greater improvements from baseline compared with zafirlukast for most efficacy measurements, including morning PEF (28.8 *vs *13.0 L/min), evening PEF (21.8 *vs *11.2 L/min), combined patient-rated symptom scores for all symptoms (-35 *vs *21%), daytime albuterol use (41 *vs *25%) and night-time salbutamol use (42% *vs *16%). Also, statistically significant differences favouring the addition of salmeterol were noted on patient-rated symptom scores for shortness of breath and chest tightness, percentage of symptom-free days, sleep symptoms, nighttime awakenings and percentage of days and nights with no albuterol use. There was no difference between the groups in symptom score for wheezing. Interestingly, the difference between salmeterol and zafirlukast was clear at week 1, but not at 4 weeks when the effect on FEV_1 _was analysed. One factor that may affect the results of this study is that there may be a randomisation bias as the proportions of patients using FP or TAA were not similar in the salmeterol and zafirlukast groups. This study was funded by the producer of salmeterol.

### Salmeterol versus zafirlukast – other literature

As a part of the above study [114], a randomised, double-blind, double-dummy parallel-group trial comparing the addition of zafirlukast (20 mg b.i.d) with the addition of salmeterol (50 μg bid) for 4 weeks in patients (n = 289) with persistent asthma, 80% of whom were on a concurrent ICS regimen has been published [115]. Both inhaled salmeterol and oral zafirlukast resulted in within-group improvements from baseline in measures of pulmonary function (morning and evening PEF and FEV_1_), asthma symptoms, and supplemental salbutamol use. Salmeterol treatment resulted in significantly greater improvements from baseline compared with zafirlukast for most efficacy measurements, including morning PEF (29.6 *vs *13.0 L/min), percentage of symptom-free days (22.2% *vs *8.8%) and percentage of days and nights with no supplemental albuterol use (30.5% *vs. *11.3%).

### Formoterol versus zafirlukast versus theophylline – other literature

An open, randomised Turkish study [116] recruited patients with moderate persistent asthma having symptoms despite the use of moderate to high doses of ICS. The patients were required to have a FEV_1 _reversibility of at least 15%. Patients (n = 64) were randomised to three different treatments budesonide (800 μg/d) plus formoterol (9 μg bid), budesonide (800 μg/d) plus zafirlukast (20 mg bid) or budesonide (800 μg/d) plus sustained-release theophylline (400 mg/d) for three months. After three months there were no between group differences in endpoints such as morning and evening PEF, PEF variability, FEV_1_, daytime or nighttime symptom scores and rescue terbutaline use. However, the addition of formoterol produced earlier improvements compared with the two other groups in criteria such as PEF variability, day- and night-time asthma symptom scores and supplemental terbutaline use. Patients in budesonide plus zafirlukast group experienced most adverse effects, but no statistical analysis was presented. The authors conclude that in patients who still have symptoms despite the treatment with ICS, the addition of any of these medications to the treatment is a logical approach and may be chosen.

### Conclusions on the comparisons between LABA, LTRA and theophylline as add-on options

LABA (salmeterol) seem to have superior efficacy as add-on therapy in persistent asthma not controlled by low to moderate doses of ICS as compared with LTRA (montelukast; four studies or zafirlukast; one study). More studies comparing the different add-on options are needed as well as studies with longer duration as the current evidence is mostly limited to follow-up period of 3 months.

## Compliance and treatment strategies

When assessing a patient with persistent asthma who is not adequately controlled by low to moderate doses of ICS:

• It is important to find out whether the patient is using the prescribed medication correctly. Poor compliance in asthma patients treated with ICS is a very common reason for treatment failure. Compliance with ICS is often less than 50% [117,118]. Oral asthma therapies may result in better compliance [119].

• Secondly, it is important to check whether the inhalation technique is adequate. Problems with the inhalation techniques are very common, especially among children and the elderly [120]. Good patient education, especially if it is self-management oriented improves health outcomes in adults with asthma [121].

• Thirdly, it is important to search for possible environmental factors, such as changes in home and working environment, hobbies and pets.

If asthma exacerbations are the dominant problem, guided self-management of asthma has been proven to be an efficient treatment strategy. In a Cochrane review [121] self-management of asthma was compared with usual care in 22 studies. Self-management reduced hospital admissions (odds ratio; OR 0.58, 95% confidence interval; CI 0.38 to 0.88), emergency room visits (OR 0.71; 95% CI 0.57–0.90), unscheduled visits to the doctor (OR 0.57; 95% CI 0.40 to 0.82), days off from work or school (OR 0.55; 95% CI 0.38 to 0.79) and nocturnal asthma (OR 0.53; 95% CI 0.39 to 0.72).

## Conclusions

Addition of formoterol or salmeterol seems to be superior as compared with the increase in the dose of the ICS in improving lung function, controlling asthma symptoms and reducing the use of rescue bronchodilator treatment. By increasing (doubling) the dose of the ICS the clinical improvement is likely to be of small magnitude. However, if frequent exacerbations are the major problem, increasing the dose of ICS may significantly help to reduce the number of exacerbations. By avoiding doses above 1000 – 1500 μg/d (budesonide and BDP) or 500 – 750 μg/d (FP) the risk of systemic adverse effects remains low. However, it should be noted that the evidence on the superiority of LABA is limited to symptomatic patients with mild to severe persistent asthma currently treated with low to moderate doses of ICS and presenting with a significant bronchodilator response. Also, addition of the LTRA montelukast or zafirlukast may improve asthma control in patients remaining symptomatic with ICS and addition of montelukast may be equal to double-dose ICS. Addition of LABA (salmeterol) seems to produce better asthma control as compared with a LTRA (montelukast or zafirlukast) whereas the long-term efficacy of LTRA (montelukast) on asthma exacerbations may be equal to LABA (salmeterol). There is evidence that addition of low-dose theophylline to the treatment regimen may be equal to doubling of the dose of ICS. However, more studies are needed to better clarify the role of leukotriene antagonists and theophylline as "add on"-therapies. For patients with inappropriate inhalation technique the value of LTRA or theophylline are especially worth considering. More studies are now needed to compare between different add-on therapies and to explore the effect of more than one add-on therapy in patients with more severe asthma as well as in those having symptoms but not significant bronchodilator response.

Another issue not addressed by these studies of large patient groups are the different responses of patients to the different add-on therapies. This needs to be studied by comparing add-on treatments in the same patients, but these studies are difficult and prolonged. In the future it may be possible to predict factors that predict the value of a particular add-on therapy in a particular patient, but the currently published studies unfortunately provide no guidance.

## Abbreviations

ACTH: corticotrophin, AMP: adenosine monophosphate, AQLQ: asthma quality of life questionnaire, BAL: bronchoalveolar lavage, BDP: beclomethasone dipropionate, ECP: eosinophil cationic protein, FEF_50_: forced expiratory flow when 50% of vital capacity has been exhaled, FENO: exhaled nitric oxide, FEV_1_: forced expiratory volume in one second, FP: fluticasone propionate, FVC: forced vital capacity, HFA: hydrofluoroalkane-134a formulation, HPA: hypothalamic-pituitary-adrenal, ICS: inhaled corticosteroid, LABA: long-acting β_2_-agonist, LTRA: leukotriene receptor antagonist, MDI: metered dose inhaler, NNH: number needed to harm, NNT: number needed to treat, PC_20_: provocative concentration causing a 20% fall in FEV_1_, PD_20_: provocative dose causing a 20% fall in FEV_1_, PEF: peak expiratory flow, TAA: triamcinolone acetonide

## Authors' contributions

HK carried out the literature searches, evaluated the studies, conceived the review and drafted the manuscript. AL, EM and PJB participated in the design and writing of the review. All authors read and approved the final manuscript.

## Supplementary Material

Additional File 1 Tables 1–7-Kankaanranta.doc contains tables 1–7 of this review. Click here for file
