# Supplementary material for: Add-on therapy options in asthma not adequately controlled by inhaled corticosteroids: a comprehensive review
Source: Respir Res. 2004 Oct 27;5(1):17. doi: 10.1186/1465-9921-5-17 (PMC528858; doi:10.1186/1465-9921-5-17)
Supplement: Additional File 1 — Tables 1–7-Kankaanranta.doc contains tables 1–7 of this review. [file 1465-9921-5-17-S1.doc]

Table 1. Inclusion criteria and characteristics of studies on the dose-dependent effects of inhaled glucocorticoids in asthma

|  | Age | Previous inhaled steroid μg/d | Classification of asthma | PEF/FEV_1_ reversibility | FEV_1_ % predicted | Other requirements for inclusion | Number of patients | Run-in (weeks) | Duration (weeks) | Oral steroid use | Other medication | Device | Study design* | Jadad  score |
| --- | --- | --- | --- | --- | --- | --- | --- | --- | --- | --- | --- | --- | --- | --- |
| Budesonide |  |  |  |  |  |  |  |  |  |  |  |  |  |  |
| Miyamoto et al. 2000 [19] | Adults | No | Mild to moderate | X | PEF 50-80% | Symptoms | 267 | 2 | 6 | No at previous 1 mo | Antihistamines allowed | Turbuhaler | R,DB,PC,PG | 3 |
| Beclomethasone dipropionate |  |  |  |  |  |  |  |  |  |  |  |  |  |  |
| Busse et al. 1999 [18] | >18 | FL, TAA, BDP 400-1000 | Moderate to severe | FEV_1_ >12% | 50-75% | X | 323 | 1-2 | 6 | No | No concurrent medications allowed | MDI | R,SB,PG | 4 |
| Fluticasone propionate |  |  |  |  |  |  |  |  |  |  |  |  |  |  |
| Dahl et al. 1993 [20] | 17-74 | BDP or BUD <1000 | Moderate | FEV_1_ >15% | 40-85 % | Symptoms and diurnal PEF variation >20% | 852 | 1 | 4 | No at previous 4 wks | Theophylline allowed, active control BDP 400 μg/d | pMDI | R, DB, PG | 3 |
| Chervinsky et al. 1994 [21] | >18 | BDP 8-16 actuations/d | Mild to moderate | X | 60-90% | X | 331 | 2 | 8 | Not during the study | Theophylline allowed | MDI | R,DB,PC,PG | 3 |
| Pearlman et al. 1997 [22] | >12 | Required | Moderate | FEV_1_ >15% | 50-80% | X | 342 | 2 | 12 | No at previous 4 wks | No concurrent medications allowed | Diskhaler | R,DB,PC,PG | 4 |
| Sheffer et al. 1996 [23] | >12 | No | Mild to moderate | FEV_1_ >15% | 45-75% | Symptoms | 307 | 1 | 12 | Not regularly at previous 2 years | No glucocorticoids or cromolyn sodium at previous 1 mo | MDI | R,DB,PC,PG | 3 |
| Wasserman et al . 1996 [24] | >12 | No | Mild to moderate | FEV_1_ >15% | 50-80% | Symptoms | 331 | 2 | 12 | No at previous 1 mo | Antihistamines, intranasal cromolyn sodium and immunotherapy allowed | Diskhaler | R,DB,PC,PG | 4 |
| Wolfe et al. 1996 [25] | >12 | Required | Moderate | X | 50-80% | X | 304 | 2 | 12 | No at previous 1 mo | Theophylline allowed | MDI | R,DB,PC,PG | 3 |
| Nathan et al. 2000 [26] | >12 | Not required | Moderate to severe | FEV_1_ >15% | 45-75% | Not required | 330 | 2 | 12 | No at previous 1 mo | Theophylline allowed | Diskus | R,DB,PC,PG | 4 |
| Mometasone furoate |  |  |  |  |  |  |  |  |  |  |  |  |  |  |
| Bernstein et al. 1999 [27] | >12 | Required | Moderate | FEV_1_ >12% | 60-90% | X | 365 | 1-2 | 12 | No at previous 1 mo | Theophylline allowed | DPI | R,DB,PC,PG | 5 |
| Bousquet et al. 2000 [28] | >12 | Required | Mild to moderate | FEV_1_ >12% | 60-90% | X | 730 | 2 | 12 | No at previous 4 wks | Theophylline allowed, active control budenoside 800 μg/d | DPI | R,EB/DB,PG | 3 |
| O'Connor et al. 2001 [29] | >12 | BDP 400-1000, BUD 400-800, FL 500-1000, FP 200-500, TAA 600-800 | Moderate | FEV_1_ >12% | 60-90% | X | 733 | 1-2 | 12 | No at previous 1 mo | No concurrent medication allowed, control FP 500 μg/d | DPI | R,DB, PG | 3 |
| **Triamcinole acetonide** |  |  |  |  |  |  |  |  |  |  |  |  |  |  |
| Welch et al. 1997 [30] | 18-64 | Not required | Moderate to severe | FEV_1_ >15% | 50-85% | X | 285 | 1-3 | 6 | X | No concurrent medications allowed | MDI | R,DB,PC,PG | 4 |
| Welch et al. 1999 [31] | >18 | Required | Moderate | FEV_1_ >15% | 50-90% | X | 514 | 1-3 | 8 | Not allowed | Intranasal medications, antibiotics and antihistamines allowed | MDI | R,DB,PC,PG | 4 |

*R= Randomized, DB= double-blind, EB=evaluator-blind, SB= single-blind, PC=placebo-controlled, PG= parallel groups,

X = not mentioned

BDP=beclomethasone dipropionate, BUD=budenoside, FL= flunisolide, FP= fluticasone propionate, MF=mometasone furoate, TAA=triamcinolone acetonide

Table 2a. The dose-dependent effects of inhaled glucocorticoids on lung function parameters and asthma symptoms.

|  |  | Morning PEF | | | Evening PEF | | | FEV_1_ | | | FVC | | |
| --- | --- | --- | --- | --- | --- | --- | --- | --- | --- | --- | --- | --- | --- |
|  | Doses studied μg/d | Dose-response | Difference between consecutive doses | Difference between highest and lowest dose | Dose-  response | Difference between consecutive doses | Difference between highest and lowest dose | Dose-  response | Difference between consecutive doses | Difference between highest and lowest dose | Dose-  response | Difference between consecutive doses | Difference between highest and lowest dose |
| Budesonide |  |  |  |  |  |  |  |  |  |  |  |  |  |
| Miyamoto et al. 2000 [19] | 200,400, 800 | Yes | No | Yes | Yes | No | No | No | No | No | * | * | * |
| **Beclomethasone dipropionate** |  |  |  |  |  |  |  |  |  |  |  |  |  |
| Busse et al. 1999 [18] | 100, 400, 800 | Yes | * | * | No | * | * | Yes | Yes | * | Yes | * | * |
| Fluticasone propionate |  |  |  |  |  |  |  |  |  |  |  |  |  |
| Dahl et al. 1993 [20] | 50,100,200, 400 | Yes | * | * | Yes | * | * | Yes | * | * | Yes | * | * |
| Chervinsky et al. 1994** [21] | 50, 200, 1000 | Yes | Yes | Yes | No | No | No | No | No | No | No | No | No |
| Pearlman et al. 1997 [22] | 100, 200, 500 | No | No | No | No | No | No | No | No | No | * | * | * |
| Sheffer et al. 1996 [23] | 50,100, 200 | No | No | No | No | No | No | No | No | No | * | * | * |
| Wasserman et al. 1996 [24] | 100, 200, 500 | No | No | No | No | No | No | No | No | No | No | No | No |
| Wolfe et al. 1996 [25] | 200, 500, 1000 | No | No | No | * | * | * | No | No | No | * | * | * |
| Nathan et al. 2000 [26] | 100, 200, 500 | Yes | Yes | Yes | Yes | No | Yes | No | No | No | * | * | * |
| Mometasone furoate |  |  |  |  |  |  |  |  |  |  |  |  |  |
| Bernstein et al. 1999 [27] | 200, 400, 800 | Yes | Yes | Yes | No | Yes | No | No | No | No | No | No | No |
| Bousquet et al. 2000 [28] | 200, 400, 800 | * | Yes | Yes | * | * | * | * | No | Yes | * | No | No |
| O'Connor et al. 2001 [29] | 200, 400, 800 | Yes | Yes | Yes | Yes | Yes | Yes | Yes | No | Yes | No | No | No |
| Triamcinolone acetonide |  |  |  |  |  |  |  |  |  |  |  |  |  |
| Welch et al. 1997 [30] | 200, 400, 800, 1600 | Yes | No | No | Yes | No | No | Yes | No | No | No | No | No |
| Welch et al. 1999 [31] | 150,300,600 | Yes | Yes | Yes | Yes | Yes | Yes | No | Yes | No | * | * | * |

| * Not studied |
| --- |
| ** Assessment of the dose-response effect based on a significant difference between the lowest and highest dose studied. |

Table 2b. The dose-dependent effects of inhaled glucocorticoids on lung function parameters and asthma symptoms.

|  |  | Symptom scores^#^ | | | Nighttime symptom score | | | β_2_-agonist use | | | HPA-axis suppression^##^ | | |
| --- | --- | --- | --- | --- | --- | --- | --- | --- | --- | --- | --- | --- | --- |
|  | Doses studied μg/d | Dose-response | Difference between consecutive doses | Difference between highest and lowest dose | Dose-response | Difference between consecutive doses | Difference between highest and lowest dose | Dose-response | Difference between consecutive doses | Difference between highest and lowest dose | Dose-response | Difference between consecutive doses | Difference between highest and lowest dose |
| Budesonide |  |  |  |  |  |  |  |  |  |  |  |  |  |
| Miyamoto et al. 2000 [19] | 200,400, 800 | Yes | No | No | Yes | No | No | * | * | * | * | * | * |
| **Beclomethasone dipropionate** |  |  |  |  |  |  |  |  |  |  |  |  |  |
| Busse et al. 1999 [18] | 100, 400, 800 | Yes | * | * | No | * | * | Yes | * | * | * | * | * |
| **Fluticasone propionate** |  |  |  |  |  |  |  |  |  |  |  |  |  |
| Dahl et al. 1993 [20] | 50,100,200, 400 | Yes | * | * | * | * | * | Yes | * | * | Yes | No | No |
| Chervinsky et al. 1994** [21] | 50, 200, 1000 | No | No | No | No | No | No | No | No | No | No | No | No |
| Pearlman et al. 1997 [22] | 100, 200, 500 | No | No | No | No | No | No | No | No | No | No | No | No |
| Sheffer et al. 1996 [23] | 50,100, 200 | No | No | No | No | No | No | No | No | No | * | * | * |
| Wasserman et al. 1996 [24] | 100, 200, 500 | No | No | No | No | No | No | No | No | No | No | No | No |
| Wolfe et al. 1996 [25] | 200, 500, 1000 | No | No | No | No | No | No | No | No | No | No | No | No |
| Nathan et al. 2000 [26] | 100, 200, 500 | No | No | No | No | No | No | No | Yes | No | No | No | No |
| **Mometasone furoate** |  |  |  |  |  |  |  |  |  |  |  |  |  |
| Bernstein et al. 1999 [27] | 200, 400, 800 | No | No | No | No | No | No | No | No | No | No | No | No |
| Bousquet et al. 2000 [28] | 200, 400, 800 | * | No | Yes | * | No | No | * | No | No | No | No | No |
| O'Connor et al. 2001 [29] | 200, 400, 800 | No | No | No | No | No | No | No | Yes | No | * | * | * |
| **Triamcinolone acetonide** |  |  |  |  |  |  |  |  |  |  |  |  |  |
| Welch et al. 1997 [30] | 200, 400, 800, 1600 | Yes | No | No | Yes | * | * | Yes | No | No | * | * | * |
| Welch et al. 1999 [31] | 150,300,600 | Yes | No | Yes | Yes | No | Yes | Yes | Yes | Yes | * | * | * |

| ^#^ Mainly daytime or total symptom score if analyzed. In case either of those is not reported, this point is marked "Yes" if one of the reported symptom scores shows dose-response. |
| --- |
| ^##^ As measured as an effect either on morning plasma/serum cortisol, urinary excretion of cortisol products or suppression of cortisol levels in cosyntropin stimulation test. |
| * Not studied |
| ** Assessment of the dose-response effect based on a significant difference between the lowest and highest dose studied. |

| Table 3. Inclusion criteria and basic characteristics of patients in studies comparing the addition of long-acting β_2_-agonist withthe increase in the dose of inhaled glucocorticoid |
| --- |

|  | Age | Previous inhaled steroid μg/d | PEF/FEV_1_ reversibility | Diurnal/ period PEFR variation | Absolute lung function as % predicted | Symptoms or score# | Oral steroid use | Rescue therapy | Exacerbation | Other medication | Jadad score |
| --- | --- | --- | --- | --- | --- | --- | --- | --- | --- | --- | --- |
| Salmeterol |  |  |  |  |  |  |  |  |  |  |  |
| Greening et al. 1994 [70] | >18 | BDP 400 | FEV_1_/ PEF >15% | > 15% | FEV_1_ >50 % | At >4 days/ 7d period | No at previous 6 weeks | X | X | Allowed | 5 |
| Woolcock et al. 1996 [71] | >17 | BDP 800-1000 or equivalent | FEV_1_ >15% | > 15%* | FEV_1_ or PEF >50% | >2/(9)*# | No at previous 4 weeks | > 4 doses at 4 days during a 7day period* | Excluded if at previous 4 weeks | X | 4 |
| Baraniuk et al. 1999 [72] | >12 | BDP 252-672, TAA 600-1000, flunisolide 1000 | FEV_1_ >15 % | X | FEV_1_ 40-85 % | X | No at previous 4 wks | X | No during screening | Theophylline allowed | 4 |
| Condemi et al. 1999 [73] | >12 | Not required | FEV_1_ >15% | > 20%* | FEV_1_ 40-65% (or 65-85%*) | >2/(5)*# | No at previous 30 days | >4 doses/24h* | Excluded if at previous 30 days | Theophylline allowed | 4 |
| Kelsen et al. 1999 [74] | >18 | BDP 400 | FEV_1_ >12% | X | FEV_1_ 45-80% | At >3 d or nights/ 7 d period | Not during the study | at > 3 days/ 7 d period* | X | Theophylline allowed | 4 |
| Murray et al. 1999 [75] | >18 | BDP 400, TAA 800 | FEV_1_ >12% | X | FEV_1_ 45-80% | At >3 d or nights/ 7 d period | No | at > 3 days/ 7 d period* | X | Theophylline allowed | 4 |
| Van Noord et al. 1999 [76] | >18 | BDP 400-600, BUD 800-1200 | FEV_1_ >10% | > 15%* | FEV_1_ >50% | >1/(9)*# | No at previous 90 days | > 2 doses/24h* | Excluded if at previous 30 days | Methylxanthines and anticholinergics allowed | 3 |
| Vermetten et al. 1999 [77] | 18-66 | BDP 200-400 | PEF >15% | X | PEF >60% | X | Not recently | X | Not recently | No concurrent medications allowed | 3 |
| Ind et al. 2003 [78] | 16-75 | BDP 1000-1600 | PEF >17.6% | >15% | X | X | Not allowed | X | 2 exacerbations during previous year required for inclusion | Other medication continued | 4 |
| Formoterol |  |  |  |  |  |  |  |  |  |  |  |
| Pauwels et al. 1997 [67] | 18-70 | BDP <2000, BUD (pMDI)<1600, BUD (TH) <800, FP <800 | FEV_1_ >15% | X | FEV_1_ >50% | X | <3 courses/ 6 mo | X | Excluded if hospitalised during previous 6 mo | X | 5 |
| O'Byrne et al. 2001 [68] | >12 | >400 mg/d BUD or equivalent | FEV_1_ >12% | >15% | FEV_1_ >70% | X | X | > 2 doses/week | X | X | 3 |
| Lalloo et al. 2003 [69] | >18 | Any brand at 200-500 | FEV_1_ >12% | X | FEV_1_ 60-90% | X | No at previous 30 days | X | X | No concurrent medications allowed | 3 |

| X= not required or not mentioned |
| --- |
| *= optional |
| #= symptom score/(total score) |

Table 4. The design of studies comparing addition of a long acting β_2_-agonist with the increase in the dose of inhaled glucocorticoid

|  | Inhaled steroid | Baseline dose (μg/d) | Comparison dose (μg/d) | Fold difference | Device | Number of patients | Run-in (weeks) | Duration (weeks) |
| --- | --- | --- | --- | --- | --- | --- | --- | --- |
|  |  |  |  |  |  |  |  |  |
| Salmeterol |  |  |  |  |  |  |  |  |
| Greening et al. 1994 [70] | BDP | 400 | 1000 | 2.5 | MDI | 426 | 2 | 26 |
| Woolcock et al. 1996 [71] | BDP | 1000 | 2000 | 2 | MDI | 494 | 1-5 | 24 |
| Baraniuk et al. 1999 [72] | FP | 200 | 500 | 2.5 | MDI | 454 | 2 | 12 |
| Condemi et al. 1999 [73] | FP | 200 | 500 | 2.5 | MDI | 437 | 2-4 | 24 |
| Kelsen et al. 1999 [74] | BDP | 400 | 800 | 2 | MDI | 483 | 2 | 24 |
| Murray et al. 1999 [75] | BDP | 200 | 400 | 2 | MDI | 514 | 2 | 24 |
| Van Noord et al. 1999 [76] | FP | 200 (LD) | 400 (LD) | 2 | Diskhaler | 60 | 4 | 12 |
|  |  | 500 (HD) | 1000 (HD) | 2 | Diskhaler | 214 | 4 | 12 |
| Vermetten et al. 1999 [77] | BDP | 200-400 | 800 | 2-4 | Diskhaler | 233 | 2 | 12 |
| Ind et al. 2003 [78] | FP | 500 | 1000 | 2 | MDI | 336 | 4 | 24 |
| Formoterol |  |  |  |  |  |  |  |  |
| Pauwels et al. 1997 [67] | BUD | 200 | 800 | 4 | Turbuhaler | 852 | 4 | 52 |
| O'Byrne et al. 2001 [68] | BUD | 200 | 400 | 2 | Turbuhaler | 1272 | 4 | 52 |
| Lalloo et al. 2003 [69] | BUD | 200 | 400 | 2 | Turbuhaler | 467 | 2 | 12 |

Table 5. The effect of addition of a long-acting β_2_-agonist as compared with increase in the dose of inhaled steroid on mean morning PEF and FEV_1_

|  |  |  | Mean morning PEF (L/min) | | |  |  |  | Mean FEV_1_ (L) | | |  |
| --- | --- | --- | --- | --- | --- | --- | --- | --- | --- | --- | --- | --- |
|  |  | Change from baseline at endpoint | | | |  |  |  | Change from baseline at endpoint | | |  |
|  | Baseline | Lower dose ICS + LABA | | Higher dose ICS | Difference | P-value |  | Baseline | Lower dose ICS + LABA | Higher dose ICS | Difference | P-value |
| Salmeterol |  |  | |  |  |  |  |  |  |  |  |  |
| Greening et al. 1994 [70] | 339-349 | 28 | | 7 | 21 | <0.01 |  | NR | NR | NR | NR | NR |
| Woolcock et al. 1996** [71] | 3.81-3.88 | 47 | | 16 | 31 | 0.005 |  | 2.31-2.48 | NR | NR | NR | <0.05 |
| Baraniuk et al. 1999** [72] | 344-361 | 58 | | 47 | 11 | ns |  | 2.12-2.14 | 0.58 | 0.48 | 0.1 | <0.033 |
| Condemi et al. 1999** [73] | 363-363 | 46.5 | | 23.8 | 22.7 | <0.001 |  | 2.12-2.14 | 0.43 | 0.33 | 0.1 | 0.013 |
| Kelsen et al. 1999 [74] | 388-390 | 47 | | 23 | 24 | <0.001 |  | NR | 0.35 | 0.26 | 0.09 | >0.05 |
| Murray et al. 1999 [75] | 381-390 | 49.4 | | 31 | 18.4 | <0.05 |  | 2.30-2.31 | 0.36 | 0.23 | 0.13 | <0.05 |
| Van Noord et al. 1999** [76] | 348-358 | NR | | NR | 6.6 | 0.42 |  | 2.33-2.34 | NR | NR | 0.05 | 0.18 |
| Vermetten et al. 1999** [77] | 390-404 | NR | | NR | NR | >0.05 |  | NR | NR | NR | NR | NR |
| Ind et al. 2003 [78] | 347-357 | 42 | | 16.5 | 25.5 | <0.001 |  | 2.3-2.4 | NR | NR | NR | NR |
| Formoterol |  |  | |  |  |  |  |  |  |  |  |  |
| Pauwels et al. 1997*/** [67] | 381-399 | NR | | NR | NR | NR |  | 2.38-2.54 | NR | NR | NR | NR |
| O'Byrne et al. 2001 [68] | 412-429 | 12.9 | | 1.7 | 11.2 | 0.0015 |  | NR | NR | NR | NR | 0.015 |
| Lalloo et al. 2003 [69] | 362-362 | 16.5 | | 7.3 | 9.2 | 0.002 |  | NR | NR | NR | NR | NR |

NR= not reported or not measured

* P-values given for placebo vs. formoterol, not exactly for low-dose-BUD + formoterol vs. high-dose-BUD

**Results clearly favor the addition of LABA more at early timepoints

Table 6. The effect of addition of a long acting β_2_-agonist as compared with increase in the dose of inhaled steroid on asthma symptoms

|  |  | Difference at the endpoint of the study | | |  |
| --- | --- | --- | --- | --- | --- |
|  | Days without symptoms or Daytime Symptom Score | Nights without symptoms or Nighttime Symptom Score | Days without rescue or daytime rescue medication use | Nights without rescue or nighttime rescue mediation use | Use of rescue medication (puffs) days & nights |
| Salmeterol |  |  |  |  |  |
| Greening et al. 1994* [70] | SM=HD-BDP | SM=HD-BDP | SM=HD-BDP | SM=HD-BDP | SM=HD-BDP |
| Woolcock et al. 1996* [71] | SM>HD-BDP | SM>HD-BDP | SM>HD-BDP | SM=HD-BDP | NR |
| Baraniuk et al. 1999 [72] | SM=HD-FP | SM=HD-FP | SM>HD-FP | NR | SM>HD-FP |
| Condemi et al. 1999* [73] | SM>HD-FP | SM>HD-FP | NR | NR | SM>HD-FP |
| Kelsen et al. 1999 [74] | SM>HD-BDP | SM>HD-BDP | SM>HD-BDP | SM>HD-BDP | NR |
| Murray et al. 1999 [75] | SM>HD-BDP | SM=HD-BDP | SM>HD-BDP | SM>HD-BDP | NR |
| Van Noord et al. 1999 [76] | SM>HD-FP | SM=HD-FP | SM>HD-FP | SM>HD-FP | NR |
| Vermetten et al. 1999 [77] | SM=HD-BDP | SM=HD-BDP | SM>HD-BDP | SM=HD-BDP | NR |
| Ind et al. 2003 [78] | SM>HD-FP | SM>HD-FP | SM>HD-FP | SM>HD-FP | NR |
|  |  |  |  |  |  |
| Formoterol |  |  |  |  |  |
| Pauwels et al. 1997** [67] | FORM>HD-BUD | FORM>HD-BUD | FORM>HD-BUD | FORM=HD-BUD | FORM>HD-BUD |
| O'Byrne et al. 2001 [68] | FORM=HD-BUD | FORM=HD-BUD | NR | NR | FORM=HD-BUD |
| Lalloo et al. 2003 [69] | FORM>HD-BUD | FORM>HD-BUD | NR | NR | FORM>HD-BUD |

NR= not reported

*Results significantly favor SM at early timepoints

**No statistical comparison made between FORM and HD-BUD

FORM= formoterol

SM= salmeterol

HD-BDP= High-dose beclomethasone dipropionate

HD-FP= High-dose fluticasone propionate

HD-BUD= High-dose budesonide

Table 7. The effect of addition of a long-acting β_2_-agonist as compared with increase in the dose of inhaled steroid on asthma exacerbations

|  | Patients with exacerbations (%)* | | | | Yearly rate of exacerbation***  no/patient/year | | | | Withdrawn if exacerbations |  |
| --- | --- | --- | --- | --- | --- | --- | --- | --- | --- | --- |
|  | Any (mild to severe) | | Moderate or severe | | Mild | | Severe** | |  |  |
| Salmeterol | High-dose steroid | Low-dose steroid + LABA | High-dose steroid | Low-dose steroid + LABA | High-dose steroid | Low-dose steroid + LABA | High-dose steroid | Low-dose steroid + LABA |  |  |
| Greening et al. 1994 [70] | 33 | 35 | 9 | 9 | 1.17 | 1.17 | 0.24 | 0.34 | No |  |
| Woolcock et al. 1996 [71] | 20 | 20 | 17 | 16 | NR | NR | NR | NR | No |  |
| Baraniuk et al. 1999 [72] | NR | NR | NR | NR | NR | NR | NR | NR | No |  |
| Condemi et al. 1999 [73] | 14 | 10 | 12 | 9 | NR | NR | NR | NR | Yes if > 2 |  |
| Kelsen et al. 1999 [74] | 17 | 15 | 11 | 8 | NR | NR | 0.48 | 0.44 | Possibly |  |
| Murray et al. 1999 [75] | 18 | 17 | NR | NR | NR | NR | NR | NR | Yes if >3 courses or lasting >14d |  |
| Van Noord et al. 1999 [76] | 7 | 0 | NR | NR | NR | NR | NR | NR | No |  |
|  | 12 | 14 | NR | NR | NR | NR | NR | NR | No |  |
| Vermetten et al. 1999 [77] | 14 | 8 | NR | NR | NR | NR | NR | NR | Yes if > 1 |  |
| Ind et al. 2003 [78] | NR | NR | 31 | 27 | NR | NR | 0.16 | 0.05 | No |  |
|  |  |  |  |  |  |  |  |  |  |  |
| Formoterol | Steroid | +LABA | Steroid | + LABA | Steroid | +LABA | Steroid | +LABA |  |  |
| Pauwels et al. 1997 [67] |  |  |  |  |  |  |  |  |  |  |
| BUD 200 μg/d | NR | NR | 38.6 | 29.7 | 35.4 | 21.3 | 0.91 | 0.67 | >3 severe/3 mo or total >5 severe ones |  |
| BUD 800 μg/d | NR | NR | 28.2 | 19.2 | 22.3 | 13.4 | 0.46 | 0.34 |  |  |
| O'Byrne et al. 2001 [68] |  |  |  |  |  |  |  |  |  |  |
| BUD 200 μg/d | NR | NR | NR | NR | NR | NR | 0.92 | 0.56 | >3 severe/3 mo or total >5 severe ones |  |
| BUD 400 μg/d | NR | NR | NR | NR | NR | NR | 0.96 | 0.36 |  |  |
| Lalloo et al. [69] | 57 | 48 | NR | NR | 2.48 | 2.07 | NR | NR | No |  |

*In case data was not found from original publications, percentages were adopted from a meta-analysis (Shrewsbury et al. [72])

**As classified by authors as severe or those requiring oral glucocorticoids (variably considered as moderate or severe).

***In case yearly rates were not given in the original publications, they were calculated from the data available.
